# X-ray microtomography imaging of craniofacial hard tissues in selected reptile species with different types of dentition

**DOI:** 10.1093/gigascience/giac016

**Published:** 2022-03-07

**Authors:** Michaela Kavková, Marie Šulcová, Tomáš Zikmund, Martin Pyszko, Jozef Kaiser, Marcela Buchtová

**Affiliations:** Central European Institute of Technology, Brno University of Technology, 616 69 Brno, Czech Republic; Department of Experimental Biology, Faculty of Science, Masaryk University, 612 42 Brno, Czech Republic; Laboratory of Molecular Morphogenesis, Institute of Animal Physiology and Genetics, v.v.i., Czech Academy of Sciences, 602 00 Brno, Czech Republic; Central European Institute of Technology, Brno University of Technology, 616 69 Brno, Czech Republic; Department of Anatomy, Histology and Embryology, Faculty of Veterinary Medicine, University of Veterinary Sciences Brno, 625 00 Brno, Czech Republic; Central European Institute of Technology, Brno University of Technology, 616 69 Brno, Czech Republic; Department of Experimental Biology, Faculty of Science, Masaryk University, 612 42 Brno, Czech Republic; Laboratory of Molecular Morphogenesis, Institute of Animal Physiology and Genetics, v.v.i., Czech Academy of Sciences, 602 00 Brno, Czech Republic

**Keywords:** micro-CT, 3D imaging, reptiles, tooth-bone attachment, skull, craniofacial bones, tooth replacement

## Abstract

**Background:**

Reptiles exhibit a large heterogeneity in teeth morphology. The main variability comprises the different tooth shape, the type of tooth attachment to the underlying bone, or the ability to replace the teeth.

**Findings:**

Here, we provide full datasets of microtomography scans and 3D models of reptilian dentitions and skulls. We selected representative species for each of 9 reptilian families on the basis of their characteristic dental features. Because there are ≥4 different types of tooth-bone attachments, ranging from the mammalian-like thecodont attachment found in crocodilians to the simple acrodont implantation observed in some lizards, we aimed to evaluate species with different types of tooth-bone attachments. Moreover, another interesting feature varying in reptilian species is the complexity of tooth shape or the number of tooth generations, which can be associated with the type of tooth attachment to the jawbone. Therefore, selected model species also include animals with distinct tooth morphology along the jaw or different number of tooth generations. The development of tooth attachment and relationship of the tooth to the jaw can be further analysed in detail on a large collection of pre-hatching stages of chameleon. Next, we introduce different possibilities for how these datasets can be further used to study tooth-bone relationships or tooth morphology in 3D space. Moreover, these datasets can be valuable for additional morphological and morphometric analyses of reptilian skulls or their individually segmented skeletal elements.

**Conclusions:**

Our collection of microcomputed tomography scans can bring new insight into dental or skeletal research. The broad selection of reptilian species, together with their unique dental features and high quality of these scans including complete series of developmental stages of our model species and provide large opportunities for their reuse. Scans can be further used for virtual reality, 3D printing, or in education.

## Background

Teeth are composed from the hardest tissues in the body of all living and extinct animals. This tissue is resistant against external intervention; therefore, its structure is sometimes the only entity that persists long enough to become useful for paleontologists to describe each fossil and place samples in the context of other extinct animals. This organ exhibits an extensive heterogeneity reflecting animal lifestyle. In herbivores, the structure of the skull including teeth is adapted to improve the grinding of plant food. In contrast, the shape of teeth in carnivorous predators is designed for the capture and processing of the prey's flesh [1].

Large heterogeneity in tooth shapes and, more importantly, the way the dentition is placed in the jaw was found especially in reptiles [2, 3]. There are ≥4 distinct types of tooth-bone attachments, ranging from mammalian-like thecodont implantation, observed in crocodiles, to the simpler acrodont type found in some lizards [4]. Tooth-bone implantation can also exhibit region-specific character across the jaw. In bearded dragons (*Pogona vitticeps*), 2 types (acrodont and pleurodont) of tooth-bone attachments are present within the jaw, with the pleurodont type located in the rostral area and the acrodont in the caudal part of the jaw [5]. Specialized complex tissue serving as an adhesive component connecting the jawbone and teeth in monitor lizards is called “plicidentin” [6].

The way the tooth is placed in the jawbone also reflects the number of tooth generations that can be initiated in certain species. Tooth replacement represents another interesting feature varying in reptiles. In chameleons, which are strictly monophydont with only 1 tooth generation, teeth are firmly attached to the jawbone, and if the tooth is lost owing to injury or during a fight, the jawbone is extensively damaged [7]. Also, the whole process of odontogenesis across individual developmental stages is peculiar, especially because of asymmetrically developing tooth germ giving rise to symmetrically ankylosed teeth [8]. However, most reptiles (e.g., geckos, *Anolis*, skinks, *Ameiva*, iguanas, and snakes) possess an unlimited and lifelong supply of new tooth generations. The type of tooth attachment is less robust in these species to ensure the constant loss of functional teeth and growth of the replacement teeth [2, 9–12].

Here, we provide full datasets of our microcomputed tomography (micro-CT) scans from various reptilian species, which can be further used for comparative morphology of the different types of tooth-bone attachments in selected representatives encompassing key species with distinct dental features. The provided datasets are also suitable for the evaluation of tooth shape differences between species or across the jaws, dental replacement patterns, to determine the facial and cranial bone structure in these species, or to analyse a large spectrum of morphometric parameters in 2 or 3 dimensions across reptiles. Moreover, this dataset is enlarged on a wide range of chameleon embryos of different ages to expand the amount of data available for further analysis such as the progression of reptilian odontogenesis with emphasis on changes in morphology of the teeth and skeletal elements with the possibility of evaluating how their relationship changes in time.

## Sampling Strategy

For this study, we enlarged the list of scanned model species, which have been used for the purpose of our previously published studies focused on tooth development [7, 8, 13, 14], by species possessing further unique features among reptiles. As a source of information for the selection of key reptilian specimens, we used a detailed review discussing the different aspects of the tooth to bone relationship across amniotes [2]. Therefore, different animal species were selected according to their unique type of tooth shape and tooth-bone relationship on which we are preferentially focused in our studies; however, these datasets can be used for numerous additional analyses that we also briefly introduce here.

*Anolis equestris* is a representative of Iguanidae, which possess so-called iguana-type implantation. In this type of attachment, the labial side of the tooth is attached to the high labial wall of the jaw; therefore it remains shorter. However, the lingual side of the tooth extends deeper into the jaw to contact the jawbone, which makes this type of attachment strongly asymmetrical. In terms of the replacement potential, this species is pleurodont with an unlimited number of tooth generations [11]. The shape of their teeth varies along the jaw. Rostral teeth are simple and conical and do not exhibit any morphological uniqueness. However, in the caudal area of the jaw, the tip of the teeth is larger and split into 3 cusps: 1 central and 2 lateral cusps.

Another selected species, commonly demonstrated as a typical pleurodont and polyphyodont, is *Paroedura picta*, belonging to the family of Gekkonidae. Geckos possess uniformly shaped teeth along the whole jaw. On the top of each tooth, there is deep groove, which divides the tooth tip into 2 ridges along the whole tooth.

Pleurodont dentition with homogenous tooth shape is typical for *Scincus scincus* from the Scincidae family. Similar to the gecko, there is an apparent uniformity in their tooth shape along the jaw. Teeth are sharp with a visible deep dent at their tip. There are several small teeth located on the palate. The whole skull is covered by scales, forming an armour-like structure, which had to be segmented to visualize the bones of the skull and teeth before 3D processing of micro-CT images.

*Timon lepidus* (previously called *Lacerta lepida*, Lacertidae), a lizard species commonly found in southwestern Europe, is another representative of pleurodont species. In the rostral part of the jaw, teeth exhibit simple monocuspid morphology, while in the caudal jaw area, teeth display 3 cusps on their tip, similarly to *A. equestris*. There are also distinct palatal teeth attached to the pterygoid bone.

As a transitional species bearing both pleurodont and acrodont type of the dentition, we selected *Pogona vitticeps* (Agamidae). Bearded dragons possess pleurodont teeth in the most rostral part of the jaw, which exhibit the ability of lifelong replacement. However, most of the teeth are acrodontly ankylosed to the underlying jawbone [5]. These two types of dentition are easily recognizable because the pleurodont teeth are monocuspid and sharply point to each other. In contrast, the acrodont dentition forms together with the jawbone compact structure, where caudal teeth are closely attached to each other on their lateral sides with complex shape consisting of 1 central and two lateral cusps.

Completely ankylosed teeth are found in *Chamaeleo calyptratus* (Chamaeleonidae). There is visible heterogeneity in tooth shape along the jaw, ranging from smaller teeth in the very rostral part of the jaw to tricuspid teeth in the caudal area, where teeth are also noticeably larger. A number of publications have described chameleon dentition in detail, with micro-CT used as one of the key methods [7, 8, 14]. In these studies, mostly the embryos were used to describe the process of odontogenesis. Therefore, so far, we know that chameleon teeth germs develop asymmetrically, closely recalling pleurodont implantation [8]. Also, the patterning of the tooth germ initiation is precisely coordinated, with the first calcified tooth appearing caudally followed by next teeth placed rostrally [15].

A unique type of tooth-jaw attachment was found in monitor lizards, where infoldings of plicidentin contribute to the junction between the tooth and the jaw [6]. To examine this so-called Varanus-type of implantation, we performed micro-CT scans of *Varanus beccarii* (Varanidae). The overall morphology of individual teeth in *Varanus* is rather simple and successional tooth generations grow in posterior-lingual direction to the functional teeth [11].

In *Salvator rufescens* (Teiidae), the heterogeneity of dentition includes both tooth shape and the type of tooth to bone attachment. In the rostral area, simple and sharp monocuspid teeth are located. More caudally, the simplicity of the tooth is replaced by teeth with bulbous shape, which helps with crunching the prey such as shellfish, small birds, or rodents. This type of implantation, often termed Dracaena-type, is unique because the teeth are seated in shallow sockets, which are deeper in the rostral area [11].

*Bitis gabonica* is a representative of poisonous snakes (Viperidae), with venomous teeth located in the rostral area of the upper jaw (**Model 1**). Their venomous teeth represent the longest fangs of venomous snakes, with a length of ∼5 cm. Gaboon vipers also possess simple conical teeth arranged into two rows in the upper jaw (inner—palatal and outer—maxillary row) and one row (mandibular) in the lower jaw. Teeth, including fangs, are replaced several times through life (polyphyodont dentition), and they exhibit a typical pleurodont type of attachment [16, 17].

As an example of nonvenomous snakes, we selected *Python regius* (Pythonidae), which is a constrictor lacking typical venomous teeth. Simple shaped teeth with sharp monocuspid morphology are curved caudally deeper to the python jaw; similar to vipers, they are arranged into two rows in the upper jaw and one row in the lower jaw. Their polyphyodont dentition is associated with pleurodont type of tooth attachment along the entire jaw [10, 16, 17].

Besides the squamate species, we also examined a specimen of *Caiman crocodilus* (Alligatoridae). All Crocodilians are known to possess tooth implantation resembling those in mammals, called thecodont gomphosis. In this type of dentition, teeth are situated in deep bony alveolus with the presence of bundles of both mineralized and non-mineralized periodontal ligaments mediating the junction force between the tooth and jawbone [18].

## Source of Samples

All analysed specimens originated from private breeders. Deceased animals were part of the collection of the Department of Anatomy, Histology and Embryology at the Faculty of Veterinary Medicine, University of Veterinary Sciences Brno (Brno, Czech Republic).

All embryonic stages of chameleon were obtained from a commercial breeder. Embryos were collected at 6 different developmental stages and fixed in 4% paraformaldehyde at least overnight. All manipulations followed the rules for working with live embryos as specified by the Central Commission for Animal Welfare of the Ministry of Agriculture of the Czech Republic ). All analyses were performed in accordance with the guidelines, regulations, and experimental protocols approved by the institutional and licensing committee including rules overseen by the Laboratory Animal Science Committee of the Institute of Animal Physiology and Genetics, v.v.i. (Liběchov, Czech Republic). No experiments were performed on live embryos.

## Micro-CT Scanning

Frozen reptile skulls were thawed in pure ethanol. To stabilize against motion during micro-CT scanning, all of the samples were placed in a plastic container selected to fit the sample and embedded in 1% agarose gel.

**Figure 1:**
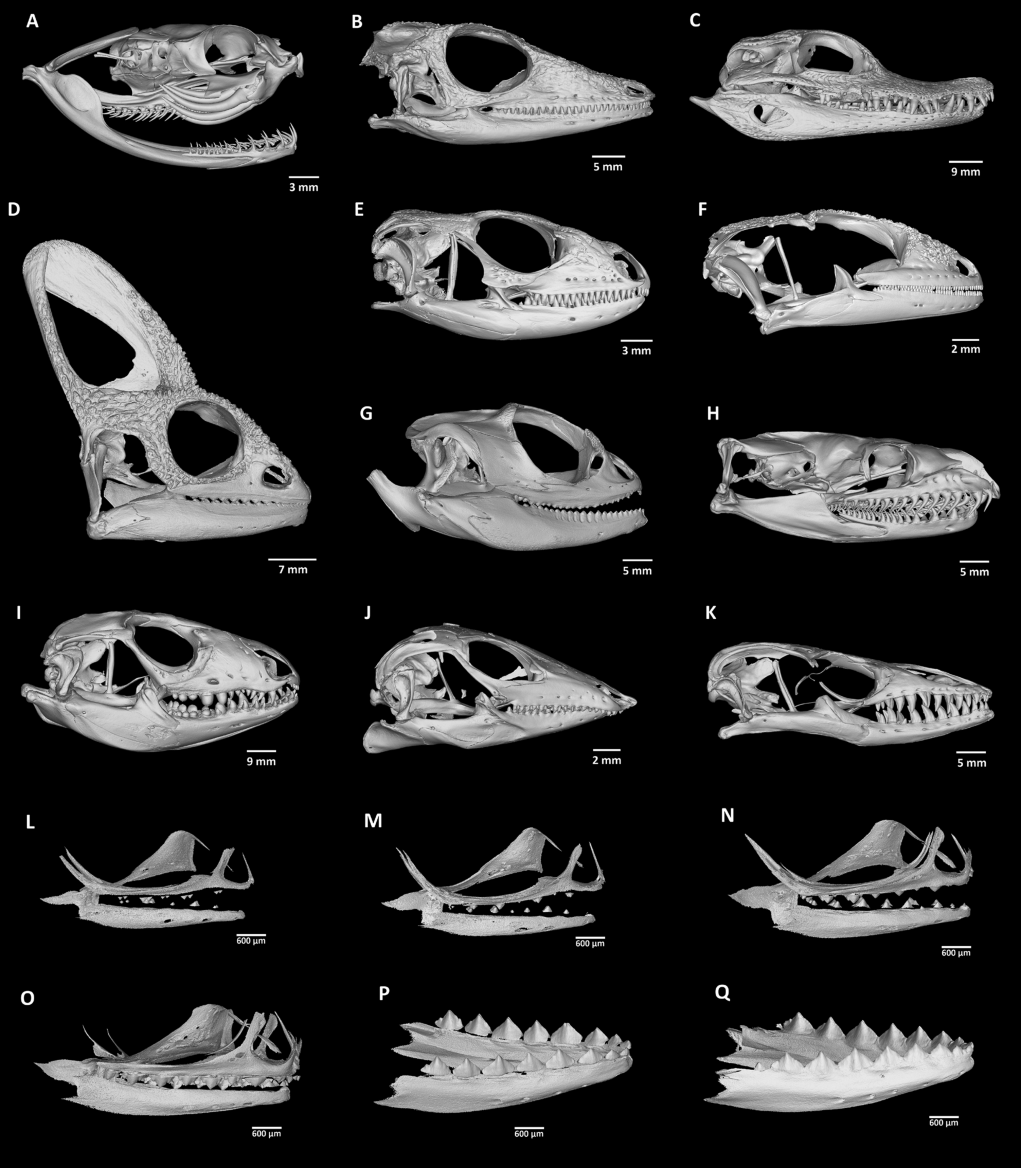
: Skull morphology of analysed reptiles (lateral view of micro-CT images). (A) *Bitis gabonica*, (B) *Anolis equestris*, (C) *Caiman crocodilus*, (D) *Chamaeleo calyptratus*, (E) *Timon lepidus*, (F) *Paroedura picta*, (G) *Pogona vitticeps*, (H) *Python regius*, (I) *Salvator rufescens*, (J) *Scincus scincus*, (K) *Varanus beccarii*, (L–Q) 6 pre-hatching stages of *Chamaeleo calyptratus*.

Eleven samples of various reptilian adult heads and 6 samples of chameleon embryo stages were selected for the aforementioned detailed micro-CT analysis of hard tissue morphology (Fig. 1, Table 1).

**Table 1:** : Scanning parameters for individual analysed animal samples

Sample	Voltage (kV)	Current (µA)	Timing (ms)	Images	Filter (mm)	Time (min)	Resolution (µm)
*Anolis equestris*	60	230	500	2,400	0.2 Al	100	24
*Bitis gabonica*	60	230	500	2,600	0.2 Al	105	19
*Caiman crocodilus*	60	230	500	2,000	0.2 Al	75	45
*Chamaeleo calyptratus*	60	230	500	2,600	0.2 Al	90	26
*Timon lepidus*	60	230	500	2,600	0.2 Al	105	18
*Paroedura picta*	60	200	750	2,500	0.2 Al	100	12.5
*Pogona vitticeps*	60	230	500	2,600	0.2 Al	110	25
*Python regius*	60	230	500	2,500	0.2 Al	105	24.5
*Salvator rufescens*	60	230	500	2,600	0.2 Al	105	48
*Scincus scincus*	60	230	500	2,100	0.2 Al	80	13
*Varanus beccarii*	60	230	500	2,600	0.2 Al	110	28

Several samples of chameleon embryos were also selected to showcase the development of the ankylotic teeth in pre-hatching stages (see list in Table 3). The micro-CT scanning was performed using laboratory system GE phoenix v|tome|x L 240 (GE Sensing & Inspection Technologies GmbH, Hürth, Germany), equipped with a 180 kV/15W maximum power nanofocus X-ray tube and high-contrast flat panel detector dynamic 41|100 (4,048 × 4,048 pixels, pixel size 100 μm). The measurements were carried out in an air-conditioned cabinet (21°C). Instrumental settings for each sample are displayed in Tables 1 and 4. The tomographic reconstruction was performed using the software GE phoenix datos|x 2.0 (GE Sensing & Inspection Technologies GmbH, Germany).

## Data Quality Control and Limitations

Because all analysed samples of adult reptile skulls were preserved prior to scanning and then handled in the same way, the main variable in data quality was the different voxel size of each dataset, owing to differing sample size. The smallest sample was the skull of *S. scincus* at 21 mm with voxel size of 13 µm, and the largest skull was *S. rufescens* at 98 mm with voxel size of 48 µm.

The difference in voxel size was directly linked to the dimensions of the sample. Considering the field of view given by the detector used and the cone beam geometry of the X-ray source of GE L240, this fact engenders a rule of thumb: the smaller the sample is, the closer to the x-ray source in the cone beam it can be placed, which finally results in smaller voxel size (higher resolution). Even though the samples display variable voxel size, this does not necessarily have to limit the analysis of the data. Larger specimens demonstrate voxel size with lower resolution than the smaller samples; however the structures being analysed are larger in these animals and are therefore easily recognizable.

In the case of the chameleon embryo samples, the embryo dimensions were mostly the same and the difference in voxel size from the smallest embryo to the largest was only 1 µm.

Samples of all reptile skulls and chameleon embryos were scanned in intact form (90% ethanol was used to fix the tissues in the adult reptile skulls, and 4% paraformaldehyde was used to fix the embryonic samples). Based on the principle of micro-CT imaging of the samples in native form without any type of staining, only the dense mineralized structures such as teeth and bones (and in the case of *S. scincus* the scales) can be visualized. Therefore, a limitation of the present dataset is a lack of information about the soft-tissue appearance and morphology in adult tissues. Because our main aim was to examine reptile skulls and tooth to bone attachment, the staining agent would affect visualization of smaller bone borders, which would become poorly distinguishable from background after counterstaining.

## Tooth Spacing Analyses by Polyline Tool

**Figure 2:**
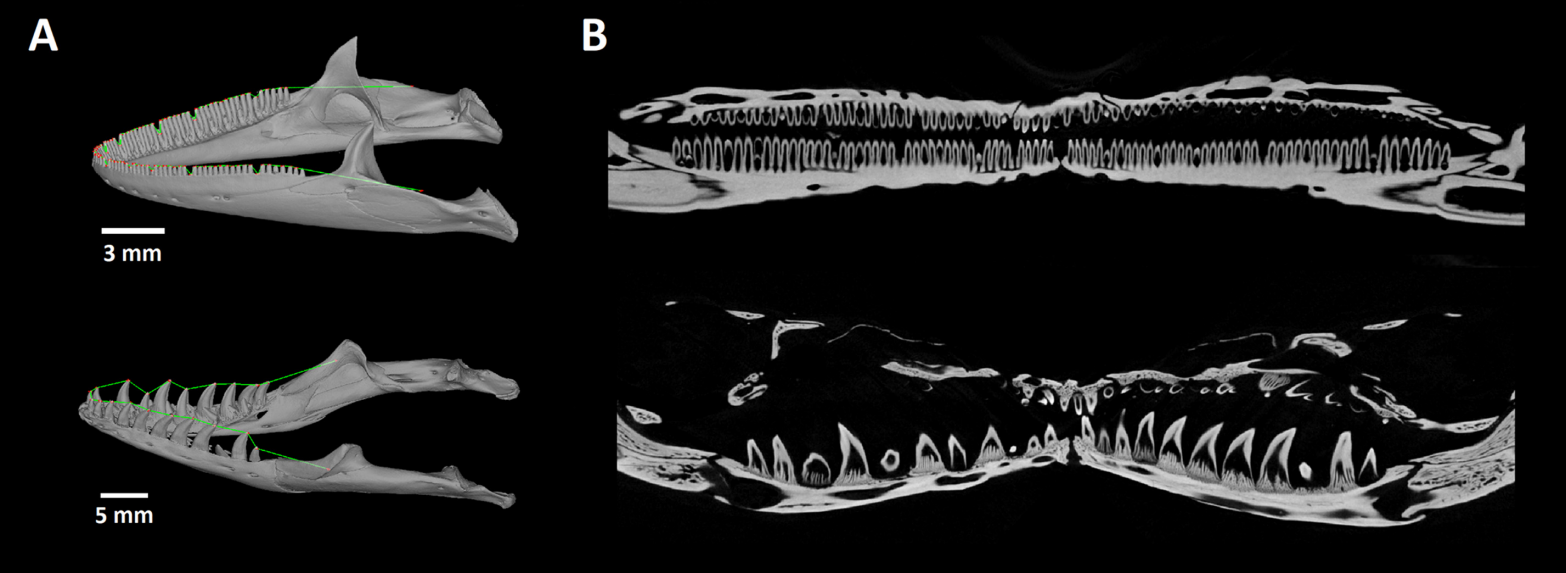
: Visualization of unrolled lower jaw using polyline tool. Examples of *Paroedura picta* and *Varanus beccarii* skulls, the design of polyline, and unrolling of the lower jaw are displayed. In the 3D render image, the points defining the polyline are presented as red dots and the produced polyline is labelled in green.

**Figure 3:**
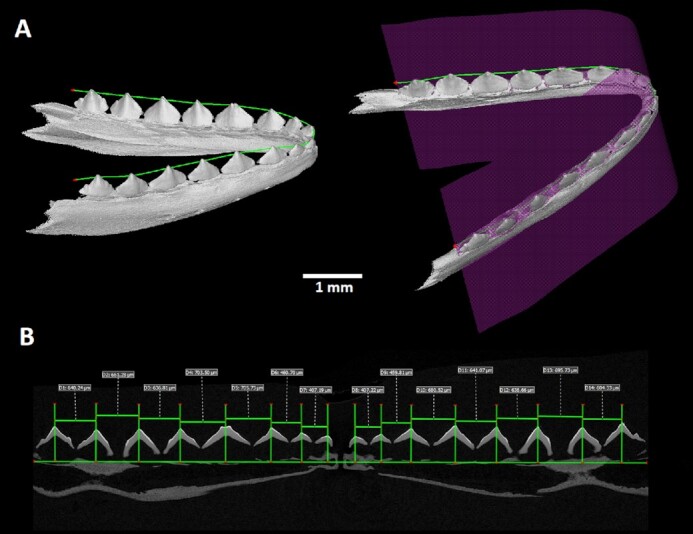
: Visualization of tooth spacing while using unrolled data of chameleon embryonic jaw. The exact placement of the unrolled section through the lower jaw is marked by the purple plane shown in the 3D render. In the unrolled image the distances between individual teeth are measured.

The polyline tool was used to create open “unrolled” images of the lower jaw (Fig. 2A). Each dataset was oriented on the basis of the main anatomical directions. In the coronal and horizontal direction, the sections were oriented to be symmetrically aligned. In the sagittal plane, where symmetry is not present, the sections were rotated to visualize as many teeth as was possible for the lower jaw. In the oriented dataset, points defining the polyline were placed on the selected tips of the teeth in the lower jaw (Fig. 2B). Finally, the unrolled images of the lower jaw were generated in a new plane defined by the polyline. The unrolled images can be used to evaluate replacement patterns (Fig. 2) or to display and determine changes in teeth spacing throughout the lower jaw as visualized in embryonic chameleon stages (Fig. 3).

The analysis of the tooth spacing in the unrolled data was slightly complicated by the fact that during the initial freezing of samples, some of them were not frozen in an optimal horizontal position, and after thawing, some structures such as the loose joint in the rostral part of the jaw in snakes were slightly bent, resulting in imperfectly oriented and unsymmetrical images. Even if in certain samples the symmetry of the unrolled images was not ideal, the required information about tooth spacing can be extracted from two separate images for each half of the jaw.

## Micro-CT Data Analysis of Tooth-Bone Attachment

**Figure 4:**
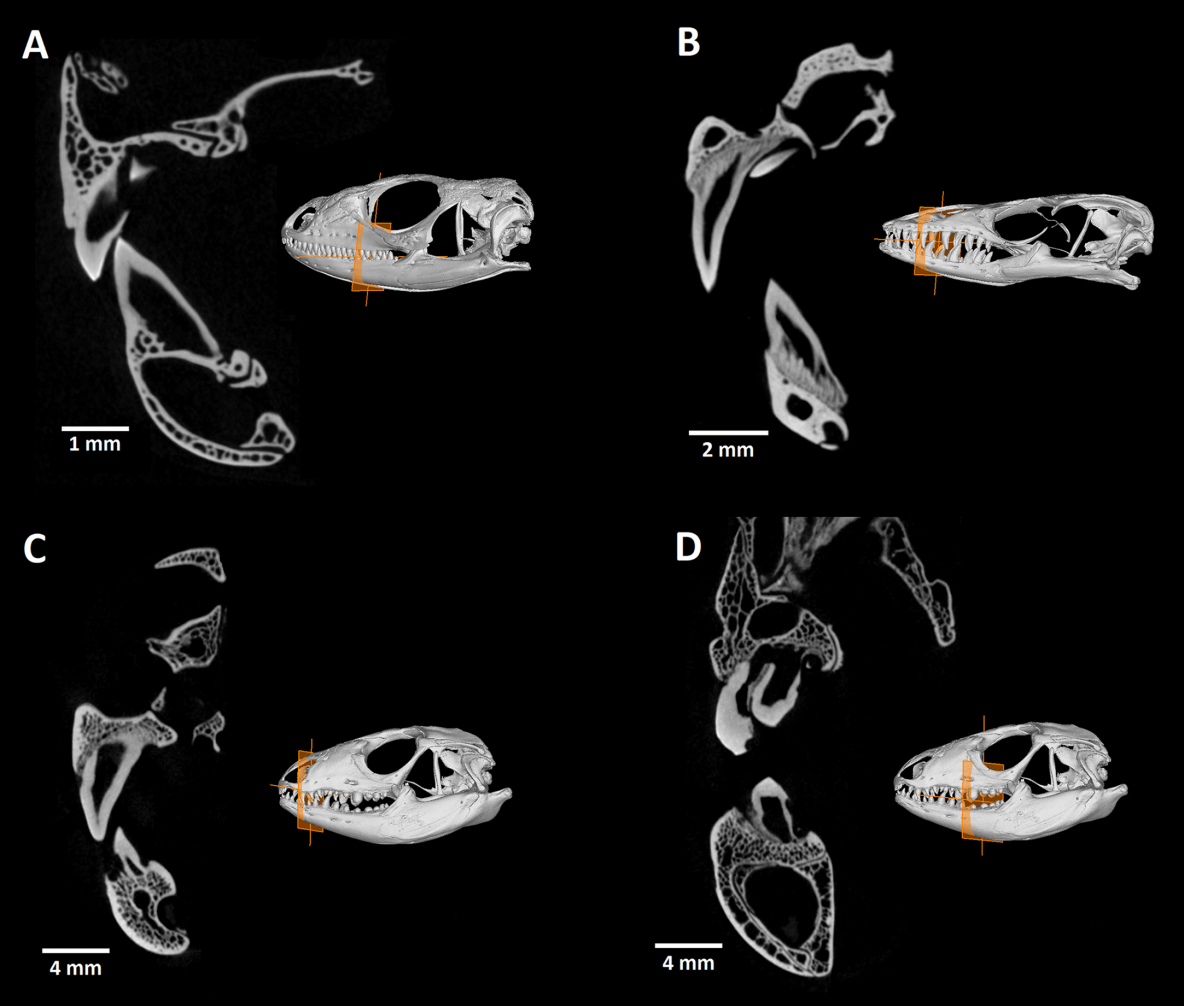
: Tooth-bone attachment in different reptiles displayed on transverse sections through the jaw. Orange plane in the 3D render of each skull demonstrates the positioning of section through the jaw. (A) In *Timon lepidus*, the most common appearance of reptilian tooth-bone interface is presented with typical asymmetrical pleurodont teeth. (B) Transverse section of *Varanus beccarii* jaw clearly displays the tissue called plicidentin, which connects the dentin of tooth to bone pedicles. (C and D) *Salvator rufescens* possesses different types and depth of tooth implantation across the jawbone.

The morphology of the tooth-bone interface and their regional differences were evaluated from micro-CT scans in VGStudio MAX 3.3 software (Volume Graphics GmbH, Germany). To achieve the optimal transverse sections through the jaw, previously generated polyline was used. The transverse sections were produced in a plane perpendicular to that of the original horizontal section. The coronal sections were oriented on the basis of the curve of the polyline (Fig. 4).

Such projections could be further used for the description of different tooth-bone relationships with regard to the individual bone appearance and the nature of tissue mediating the interface. Moreover, because the section could be placed anywhere throughout the skull, such structures as palatal teeth appearance and their distribution, temporomandibular joint morphology, or the spatial relationship among individual craniofacial bones can be studied.

## Skull Wall Thickness Analysis

Micro-CT scans can be used to evaluate hard-tissue density and thickness, which could serve as a source of valuable information for deciphering different aspects of skull anatomy, as well as their interspecies differences.

VG Studio software enables two methods of wall thickness analysis: a ray-based method and a sphere-based method. The ray-based method defines the wall thickness by searching the opposite surface by sending a measurement ray perpendicularly to the first surface. Calculated wall thickness is then defined by the shortest distance between 2 crossed rays from opposite surfaces. The sphere-based method evaluates the wall thickness by fitting spheres inside the sample in 3D space. The thickness of the analysed structure is then defined by the diameter of the fitted sphere.

**Figure 5:**
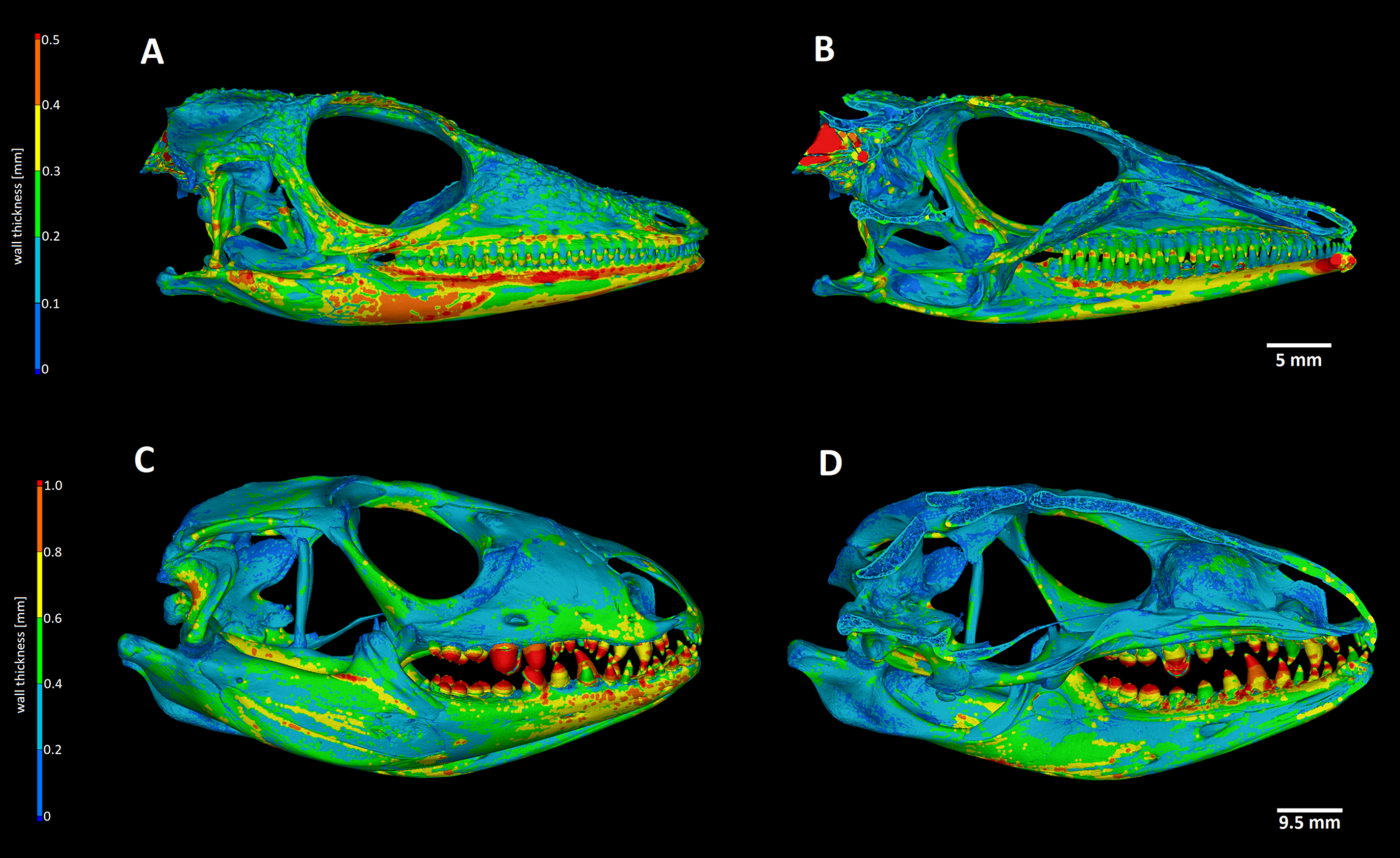
: Wall thickness analysis of reptilian teeth and adjacent jaws. (A) *Anolis equestris:* The thickest area on the anolis skull is localized along the upper part of the lower jaw, suggesting its importance as a support during food processing. (B) Sagittal section through the midline of the skull in *A. equestris*. (C) *Salvator rufescens:* In contrast to anolis, wall thickness analysis displayed the greatest tissue density in bulbous caudal teeth, which need to be strong enough to process hard materials such as shells. (D) Sagittal section through the midline of the skull of *S. rufescens*.

In the case of complex bone structure, the sphere-based measurement of wall thickness analysis is more accurate; therefore here we introduce data from this type of analysis on our selected species (Fig. 5, Supplementary Video S1). The colour scale of the wall thickness analysis displays the hard-tissue elements, which exhibit more gracile morphology, and we were able to distinguish them from reinforced elements. This information can facilitate the estimation of strains influencing specific parts of the skull leading to food-processing adaptations.

## Analysis of Single Tooth Morphology

The high resolution of the obtained micro-CT data enables precise segmentation of specific structures of the analysed sample. As an example, the teeth of several species were segmented. The process of segmentation includes the global thresholding step to define the hard tissues from background. In the following step, the object of interest (in our case the venomous tooth of *B. gabonica* and replacement tooth of *C. crocodilus*) is roughly marked by 3D brush, and by intersecting the rough model of the tooth with the defined bones, the precise model of the tooth is created.

**Figure 6:**
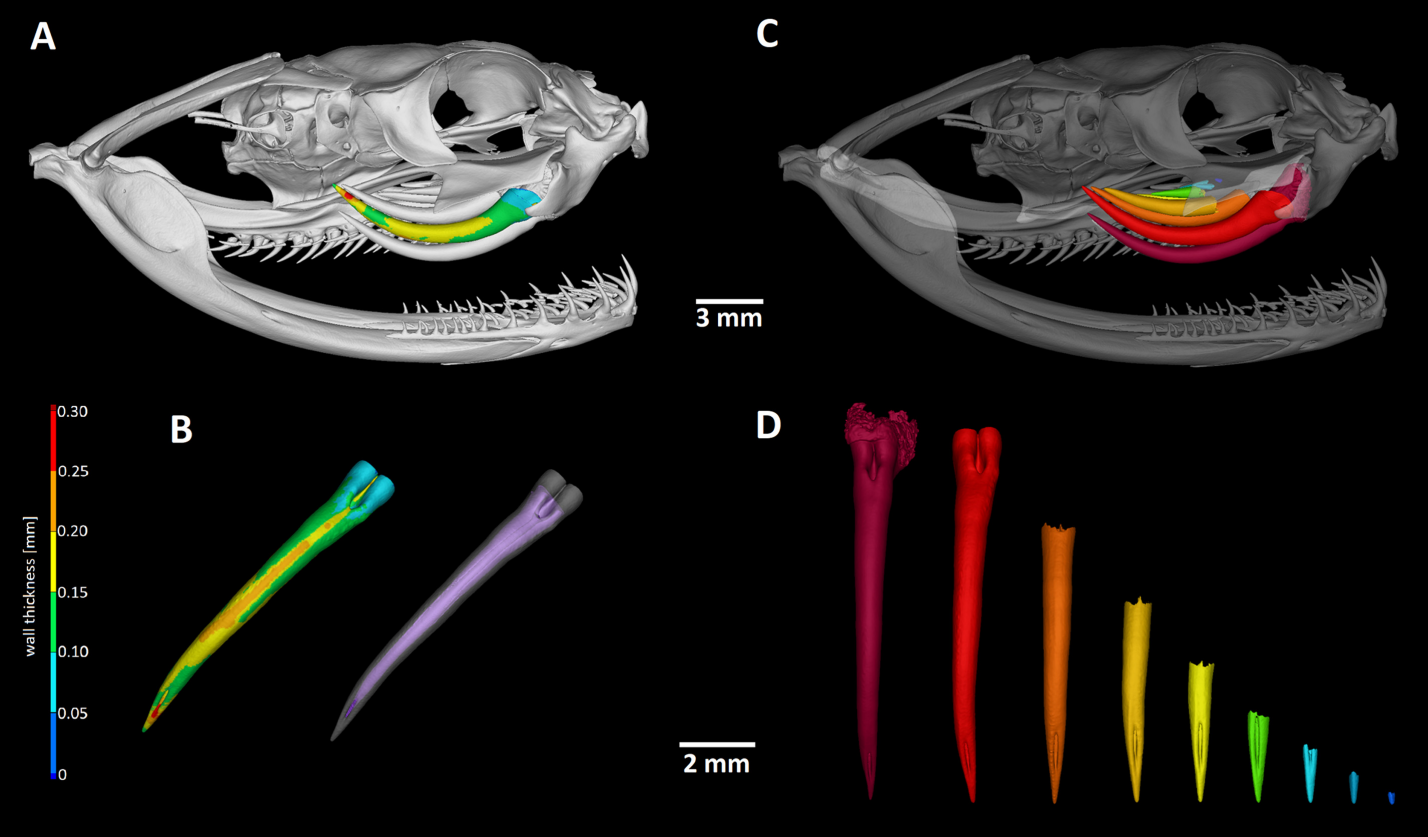
: Segmentation and analyses of individual teeth in *Bitis gabonica*. (A) The second generation of the venomous tooth on the left side of the upper jaw was selected for wall thickness analysis. (B) Detail view on the second largest segmented tooth with applied wall thickness analysis, where red colour labels the thickest area of the fang and blue the thinnest areas. The same tooth was made partially transparent to display the inner structure of the tooth. (C) All of the venomous teeth on the left side of the upper jaw were segmented to uncover replacement tooth generations. (D) Detail view on all generations of fangs visualized in different colour. Note differences in the size and position of distal openings among individual generations.

**Figure 7:**
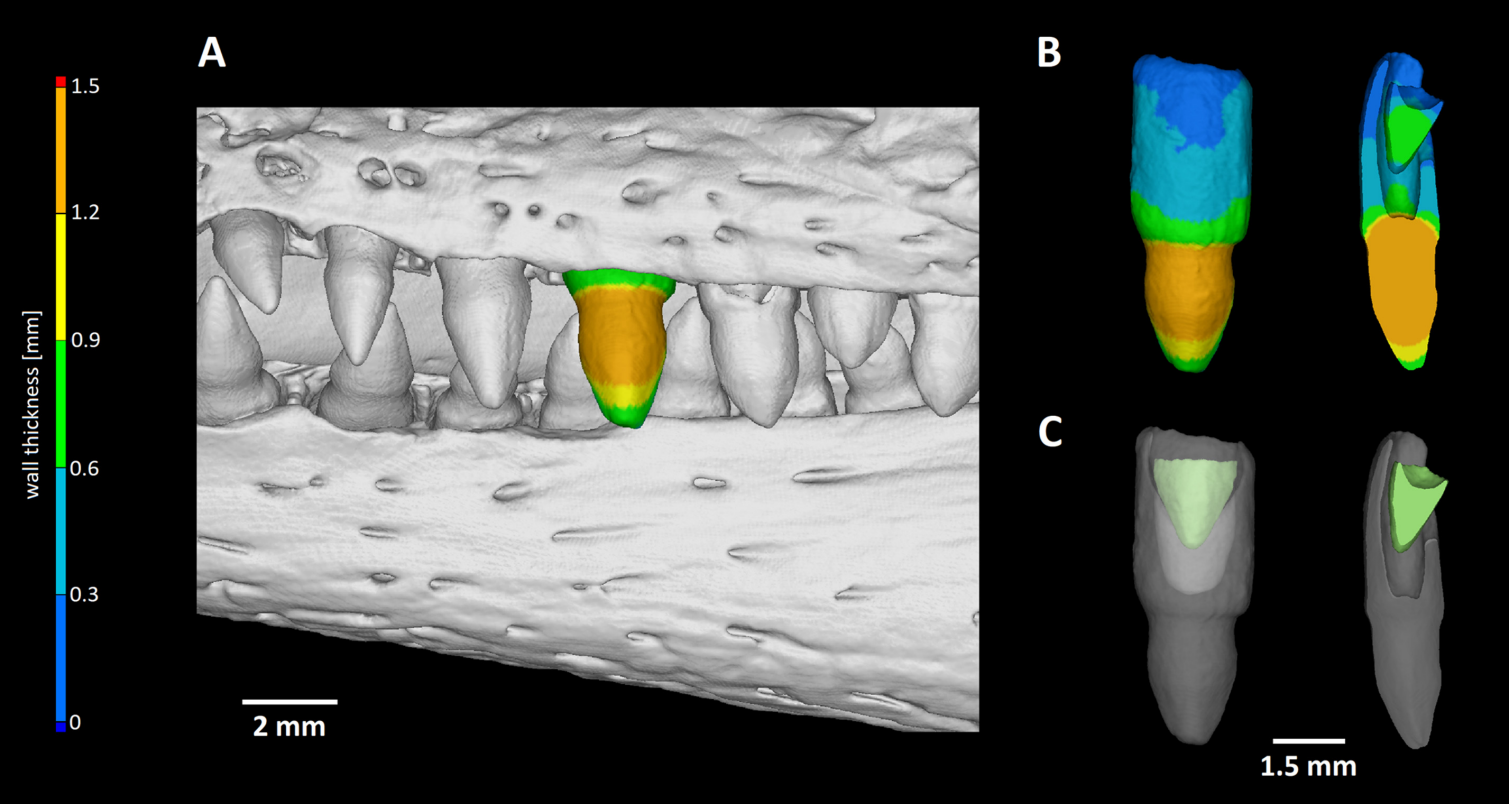
: Visualization of replacement teeth in *Caiman crocodilus*. (A) Localization of individual teeth in the jaw with one segmented tooth. For precise morphology description, wall thickness analysis was applied. Orange colour labels the thickest areas of the tooth. (B) Segmented tooth is visualized without surrounding jawbone by wall thickness analyses. Horizontal section through the analysed tooth revealed new generation of teeth located inside of functional tooth. (C) Replacement tooth is labelled in green and functional tooth is translucent to visualize the position of next generation formation.

Samples of *B. gabonica* and *C. crocodilus* were selected to display some possibilities for more detailed tooth analysis (**Model 1**,Fig. 6 and 7). These species represent different types of tooth morphology (venomous teeth in *B. gabonica* and simple conical teeth in *C. crocodilus*), as well as tooth implantation (pleurodont implantation in *B. gabonica* and thecodont implantation in *C. crocodilus*).

In the case of *B. gabonica*, venom teeth from the left side of the skull were segmented and visualized (Fig. 6, Supplementary Video S2). The volume of teeth can be easily measured (the volume of the largest visualized tooth was 3.88 mm^3^ in this sample) (Fig. 6). Transparent visualization enables us to determine dental pulp morphology and analyse its shape in individual teeth or its communication to bone marrow as we discussed previously in chameleon [7].

Micro-CT analysis of *C. crocodilus* teeth enabled us to visualize the next generation of teeth hidden in the currently employed tooth (Fig. 7, Supplementary Video S3). The shape and volume of individual teeth can be evaluated along the jaw and compared across regions. Here, the smaller tooth volume was 1.22 mm^3^ and the volume of the largest tooth was 11.44 mm^3^.

This type of analysis offers great potential for investigating a number of aspects such as precise tooth shape and morphology description, dental pulp filling and/or venomous canal formation, replacement pattern in polyphyodont species, and visualization of individual tooth generation. All of the aforementioned analyses can be applied to any craniofacial structure of interest. The only condition is previous careful segmentation of the analysed structure before undertaking further analyses.

## Palatal Tooth Analyses

**Figure 8:**
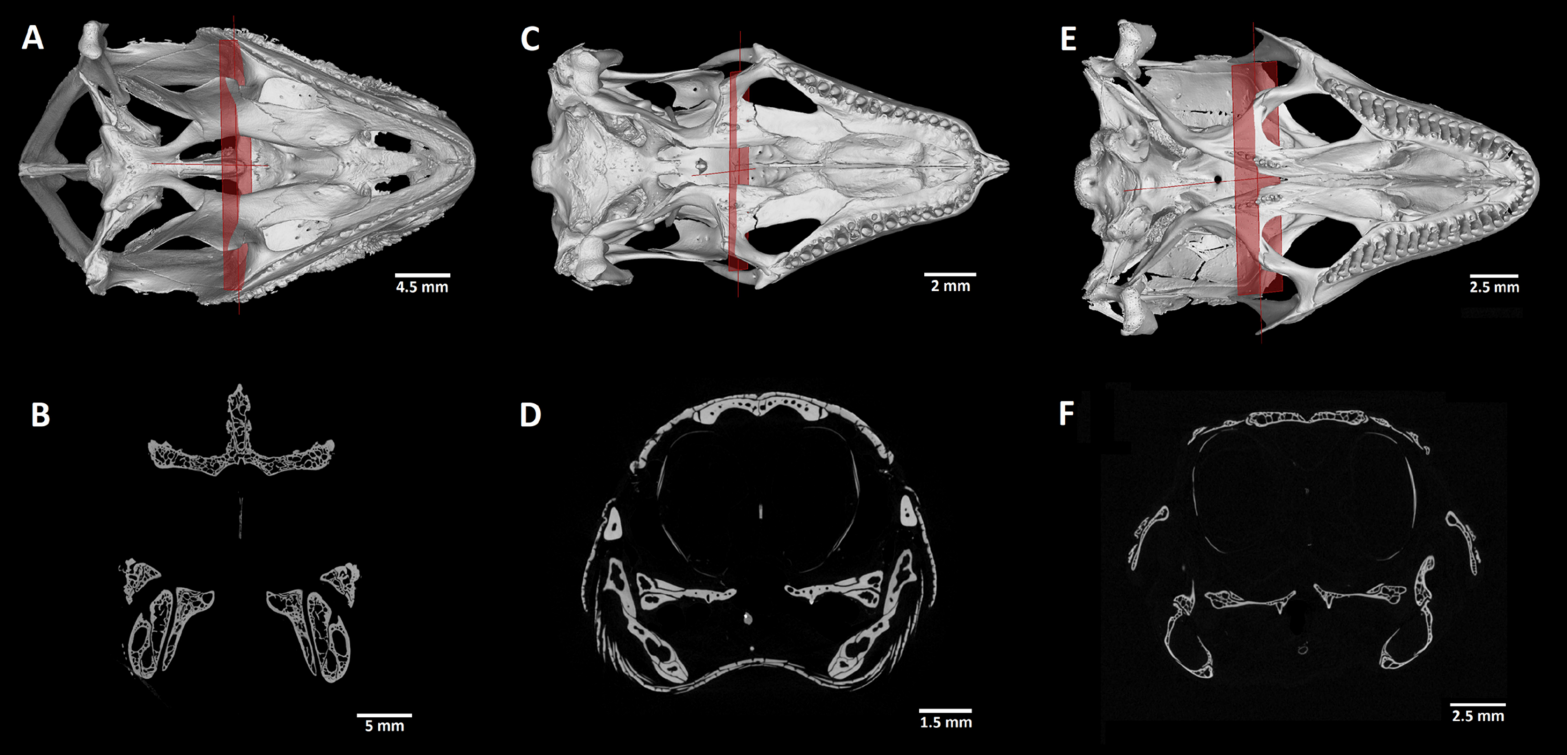
: Palatal view on the upper jaw and transverse sections through the palatal teeth. (A) *Chamaeleo calyptratus:* palatal view of 3D render image. (B) *Chamaeleo calyptratus:* transversae section through the skull defined by the red plane indicated in 3D render displays the absence of palatal teeth. (C) *Scincus scincus:* palatal view of 3D render image uncovers two pairs of palatal teeth located on the pterygoid bone. (D) *S. scincus:* transverse section through the skull defined by the red plane indicated in 3D render visualizes the palatal teeth; yellow arrow indicates the location of teeth. (E) *Timon lepidus:* palatal view of 3D render image displays long row of palatal teeth. (F) *Timon lepidus:* transverse section through the skull defined by the red plane indicated in 3D render demonstrates the palatal teeth.

The presence of palatal teeth in reptiles is diverse (Fig. 8). Some species do not bare teeth in the palatal area (chameleons), while in other species, they play an important role in food transport through the oral cavity (venomous snakes) [19]. Palatal teeth can also serve as a tool for processing of plant food or cracking hard-shell prey [20]. The overall morphology of palatal teeth is rather simple in comparison with that of marginal teeth [19]. The presence or absence of structures such as palatal teeth could also predicate evolutionary similarity across reptiles. Therefore, the present dataset could broaden this under-evaluated topic and offer valuable information for further study.

## Segmentation of Selected Craniofacial Bones

Our dataset also provides great potential in the possibility to evaluate not only dentition morphology or individual teeth but also other hard craniofacial tissues. Thus, the dataset represents a robust system that could be applied for examination of any scanned skeletal structure. Precise segmentation is crucial because once the structure in question is visibly discriminated, further evaluation of its morphology and/or the interspecies differences is much easier and more accurate. However, this tool also provides the flexibility of describing each skeletal structure in detail separately or in context with individual surrounding elements. Besides the study of interspecies differences, segmented structures could be used for study of intersex variability in skull morphology or dynamics of bone development/progress.

**Figure 9:**
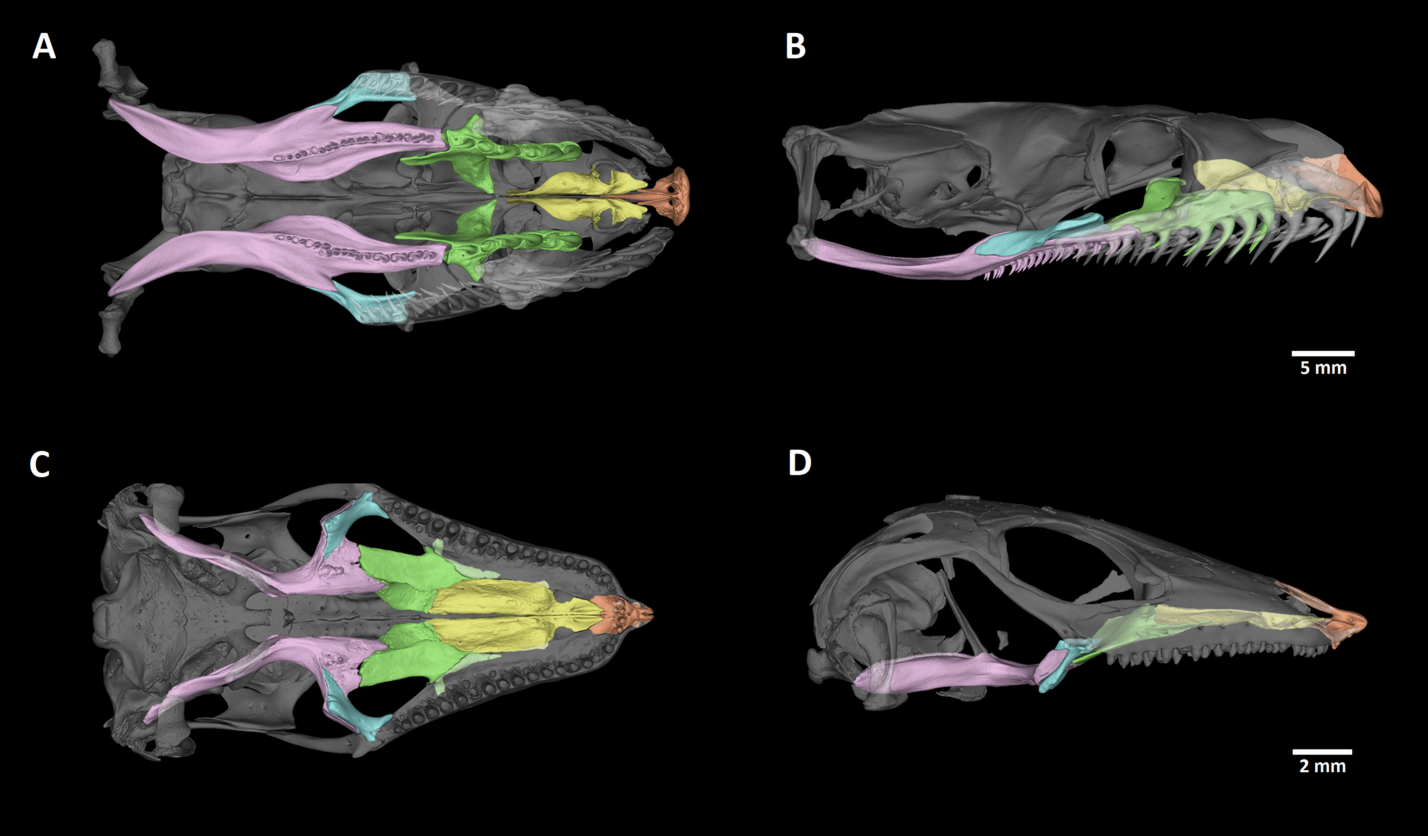
: Arrangement of bones contributing to the palate in *Python regius* and *Scincus scincus*. (A) Palatal view of the upper jaw of *Python regius*. (B) Sagittal view of the bones supporting the palate in *P. regius*. (C) Palatal view of the upper jaw of *S. scincus*. (D) Sagittal view of the bones supporting the palate in *S. scincus*. Individual skeletal elements were segmented and labelled as follows: orange: premaxillary bone; yellow: vomer; green: palatal bone; pink: pterygoid; blue: ectopterygoid. The bones of the skull surrounding the segmented palatal bones were made semi-transparent to improve the display of the analysed areas.

**Figure 10:**
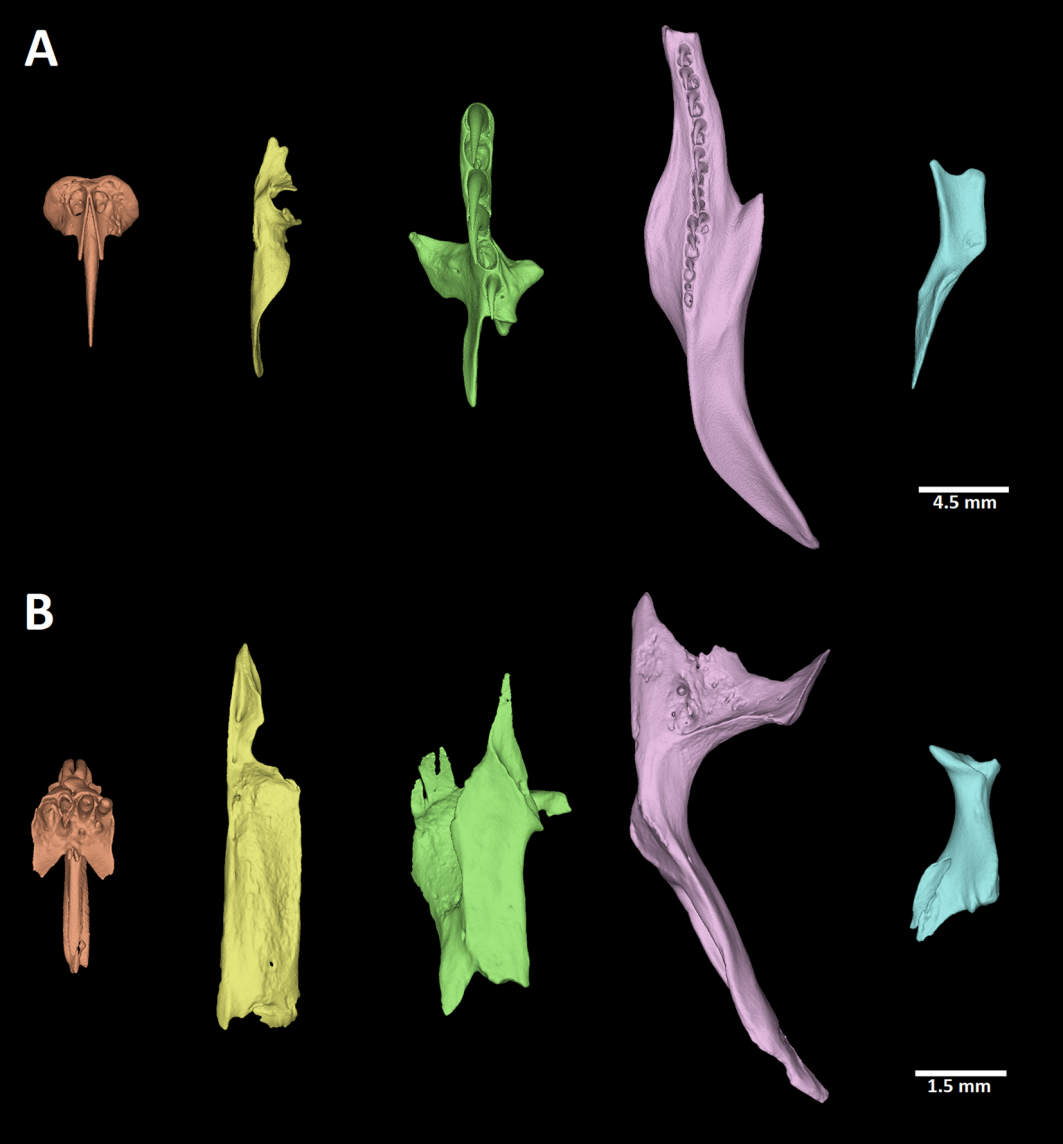
: Segmented bones contributing to the palate in selected reptiles. (A) *Python regius*, (B) *Scincus scincus*. Palatal view of individual segmented skeletal elements in both species: orange: premaxillary bone; yellow: vomer; green: palatal bone; pink: pterygoid; blue: ectopterygoid.

Here, VG Studio Max software was used to segment the craniofacial bones (Figs. 9 and 10, Supplementary Video S4). The first module used was “surface determination,” which was applied to distinguish the bones from the background. The region of interest (ROI) of all bones was determined. In the following step, the tool “3D brush” was used to mark the approximate ROI of selected bones contributing to the palate. The final step was then generation of the ROI containing only the selected bone by intersecting the ROI of all bones with the marked ROI of the bone.

## Volume Analyses of Skulls and Skeletal Elements

To make our analysis of segmented skull bones more detailed and therefore highly applicable, a volume analysis was subsequently applied. Volumes of whole skull or bones of interest can be analysed from 3D data (see examples in Table 2). Acquired data could be used for assessing interspecies differences in bone volumes, which could be then used for deciphering of evolutionary questions and other related topics.

**Table 2:** :Volumes of analysed skulls

Species	Volume (mm^3^)		
	Skull	Maxilla	Mandible
*Anolis equestris*	870	584	286
*Bitis gabonica*	232	181	51
*Caiman crocodilus*	6,409	4,108	2,301
*Chamaeleo calyptratus*	1,441	1,062	379
*Timon lepidus*	197	133	64
*Paroedura picta*	113	81	32
*Pogona vitticeps*	2,236	1,384	852
*Python regius*	1,817	1,308	509
*Salvator rufescens*	10,869	6,232	4,637
*Scincus scincus*	108	71	37
*Varanus beccarii*	1,130	757	373

**Table 3:** : List of analysed chameleon embryo samples

Sample	Date		Embryo age (days)	Weight (g)	
	Clutch laying	Embryo collection		Egg	Embryo
Stage 1 (CHM379)	12.01.2019	20.05.2019	128	1.23	0.25
Stage 2 (CHM313)	08.05.2018	31.08.2018	115	1.31	0.37
Stage 3 (CHM389)	12.01.2019	27.05.2019	135	1.77	0.4
Stage 4 (CHM397)	12.01.2019	06.06.2019	145	1.47	0.44
Stage 5 (CHM327)	21.06.2018	14.11.2018	146	1.81	0.65
Stage 6 (CHM351)	21.06.2018	10.12.2018	173	1.38	0.82

**Table 4:** : Scanning parameters for individual analysed pre-hatching embryos

Sample	Voltage (kV)	Current (µA)	Timing (ms)	Images	Filter (mm)	Time (min)	Resolution (µm)
Stage 1 (CHM379)	60	200	600	2,500	0.2 Al	105	2.5
Stage 2 (CHM313)	60	200	600	2,400	0.2 Al	105	3.5
Stage 3 (CHM389)	60	200	600	2,400	0.2 Al	105	3
Stage 4 (CHM397)	60	200	600	2,400	0.2 Al	105	3
Stage 5 (CHM327)	60	200	600	2,200	0.2 Al	85	3
Stage 6 (CHM351)	60	200	600	2,300	0.2 Al	85	3.5

## Further Biological Potential

Indisputable biological potential of the present dataset lies in the number of different species that were scanned and the quality of the scans, as well as large possibilities for subsequent analysis on the acquired data. Therefore, this dataset can be used for further studies concerning comparative ecology and evolutionary biology or morphological/anatomical topics. Here, we introduce different possibilities of data reuse potential with the main focus on craniofacial structures.

The acquired data could be helpful in the study of different types of tooth-bone attachment and implantations, which is, according to recent publications, a broadly studied topic [8, 10, 11, 21–24] that varies across species. Another interesting feature concerning teeth is the analysis of tooth spacing, which we addressed using a specific tool in VG studio Max that allows the jaw to be "unrolled" to display all the teeth at once. Micro-CT analysis also enables the process of tooth replacement to be precisely described with closer focus on the position of the next tooth generations in comparison to the first generation or deciphering of the general replacement pattern, which can be also evaluated in our dataset. Thanks to the segmentation tool, study of the poison flow throughout the venomous canal in fangs is possible, as is determination of dental pulp changes across the jaw.

Moreover, the obtained data could be used for further studies by those who are particularly interested in reptilian skull anatomy (e.g., temporomandibular joint, columella, palatal bones, or other structures) and its evolutionary differences across diverse species. These skeletal elements can be evaluated by the tool, which enables the precise segmentation of individual structure. These segmented bones could be further processed to get precise information about their shape, volume, and relationship to other surrounding structures in 3 dimensions. Furthermore, wall thickness analysis can be useful to study structural aspects of skeletal elements.

To broaden our knowledge about reptilian skull anatomy, morphology, and development, the scans of multiple chameleon embryos are provided. The entire present analysis, in combination, will provide comprehensive and detailed information about both dental and bone tissue morphology in selected model species across reptilians.

## Popularization/Educational Potential of Present Dataset

**Figure 11:**
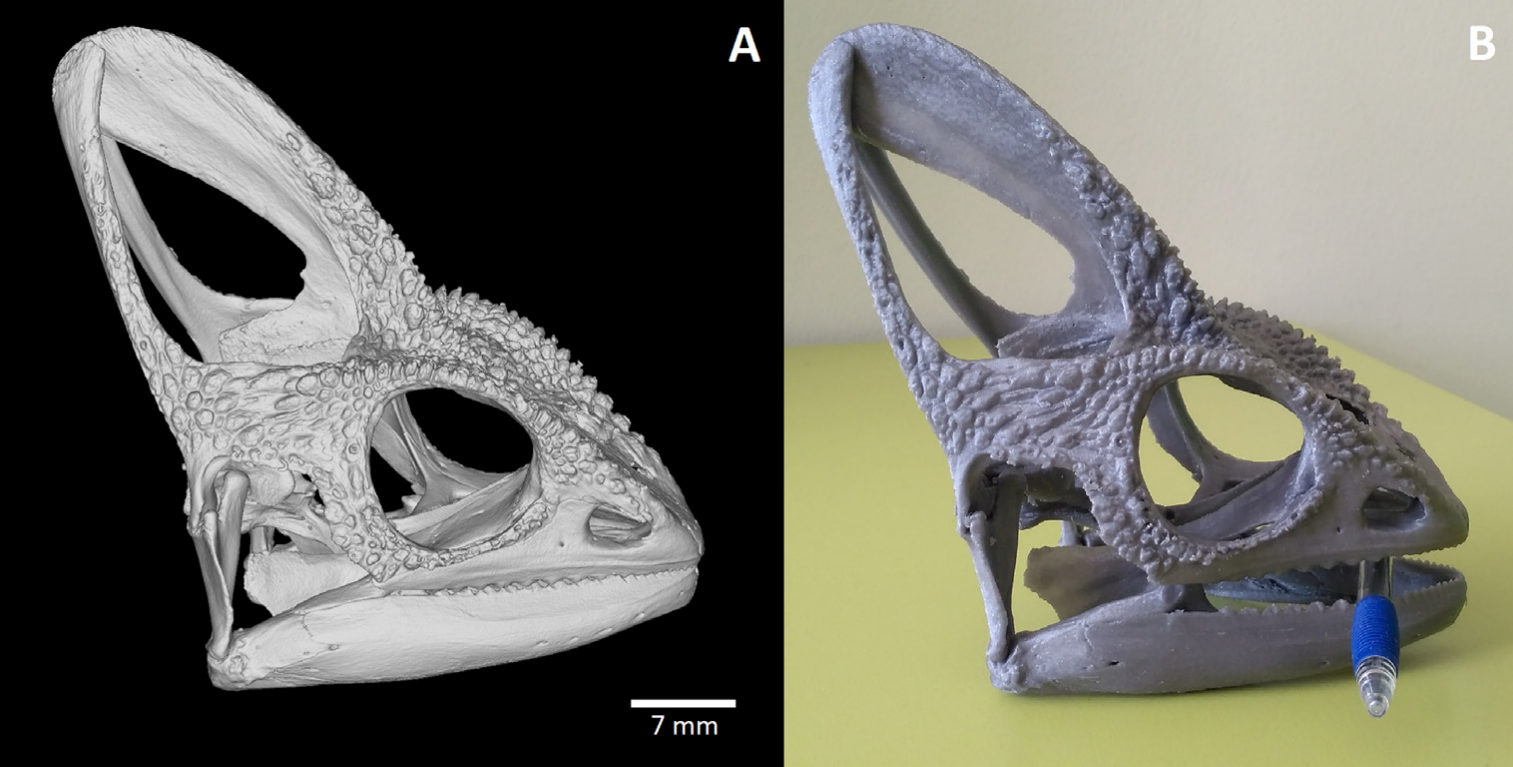
: 3D-printed skull of *Chamaeleo calyptratus* from generated stl model. (A) 3D stl model of the scanned *C. calyptratus* skull generated in VG Studio MAX, (B) 3D printed model of the skull. For the purpose of 3D printing, the generated stl model was 3.5× magnified. The PRUSA MK3S printer with the PLA filament and 0.15-mm printing layer was used to create the model; total print time was 40 hours.

The 3D-printed models of entire analysed reptilian skulls or individual segmented skeletal elements of interest can also be used as an innovative learning support for university or secondary school students. Both 3D-printed and virtual reality models could also be presented to the general public, e.g., at museums without any need to possess original skulls or taxidermy mounts (Fig. 11). Use of 3D scans for virtual reality enables not only detailed view of individual structures but also the option of walking through a skull or inside of individual skeletal elements.

**Model 1:**
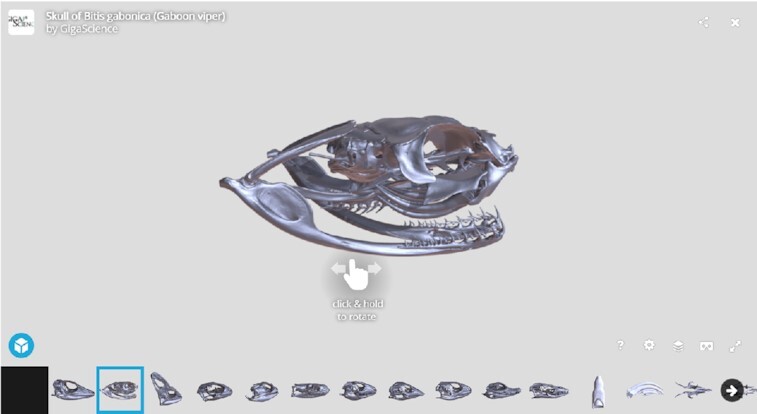
: *Bitis gabonica*. Because the 3D surface-rendered image is very large for convenient download and inspection, we provide a lower-resolution version for web-based 3D visualization in Sketchfab. This model was smoothed using Amira, with the modified version uploaded to Sketchfab. The complete Sketchfab collection is embedded in the GigaDB dataset [25] and is also available via https://sketchfab.com/GigaDB/collections/reptile-skull.

## Data Availability

Volumetric data of all scanned animals are available in 8-bit tiff stack files via the *GigaScience* database GigaDB [25]. These tiff stacks include the raw tomographic sections. The data were reduced from original 16 to 8 bit to make the data more compact to download and to make it more available for analysis, considering that the larger datasets need high computational power to even open them. The stl models of skulls were created for all analysed samples; these models are highly detailed, which makes them suitable for 3D printing at higher magnifications. Additional tiff stacks of demonstrated analyses (unrolled lower jaw) are included. Finally, videos displaying individual analyses in detail (such as wall thickness analyses, teeth or bone segmentation) or transverse sections through the head are also included. Models are also available via the Sketchfab repository https://sketchfab.com/GigaDB/collections/reptile-skull.

## Additional Files

**Supplementary Video S1:** *Salvator rufescens* in 3D view while using wall thickness analyses

**Supplementary Video S2:** 3D view of individual fang teeth in *Bitis gabonica*

**Supplementary Video S3:** 3D visualization of replacement teeth in *Caiman crocodilus*

**Supplementary Video S4:** *Scincus scincus* in 3D view with focus on segmented palatal elements

giac016_GIGA-D-21-00360_Original_Submission

giac016_GIGA-D-21-00360_Revision_1

giac016_GIGA-D-21-00360_Revision_2

giac016_Response_to_Reviewer_Comments_Revision_1

giac016_Response_to_Reviewer_Comments_Revision_2

giac016_Reviewer_1_Report_Original_Submission Anton du Plessis -- 11/26/2021 Reviewed

giac016_Reviewer_2_Report_Original_Submission Chris Armit -- 12/1/2021 Reviewed

giac016_Reviewer_2_Report_Revision_1 Chris Armit -- 12/21/2021 Reviewed

giac016_Supplemental_Files

## Abbreviations

micro-CT: microcomputed tomography; ROI: region of interest; stl: standard template library.

## Competing Interests

The authors declare that they have no competing interests.

## Funding

This study was supported by the Ministry of Health, Czech Republic (NU20-06-00189) and the CzechNanoLab Research Infrastructure supported by MEYS CR (LM2018110).

## Authors' Contributions

Data analyses: M.K.

Visualization: M.K., M.S.

Validation: T.Z.

Conceived and designed experiments: T.Z., M.B.

Grant support: M.B.

Contributed reagents and materials: M.P., J.K.

Software: T.Z., J.K.

Writing of original manuscript: M.K., M.S., M.B.

Writing—review & editing: T.Z., M.P., M.B.
