# Supplementary material for: X-ray microtomography imaging of craniofacial hard tissues in selected reptile species with different types of dentition
Source: Gigascience. 2022 Mar 7;11:giac016. doi: 10.1093/gigascience/giac016 (PMC8900495; doi:10.1093/gigascience/giac016)
Supplement: giac016_GIGA-D-21-00360_Revision_1 [file giac016_GIGA-D-21-00360_Revision_1.pdf]

GigaScience

DATA NOTE:
X-ray microtomography imaging of craniofacial hard tissues in selected reptile species with different types of dentition

--Manuscript Draft--

**Manuscript Number:**	GIGA-D-21-00360R1
**Full Title:**	DATA NOTE:<br>X-ray microtomography imaging of craniofacial hard tissues in selected reptile species with different types of dentition
**Article Type:**	Data Note
**Funding Information:**	Ministerstvo Zdravotnictví Ceské Republiky (NU20-06-00189/2020) — Dr. Marcela Buchtova
**Abstract:**	Background: Reptiles exhibit a large heterogeneity in the teeth morphology. The main variability is comprised by the different tooth shape, the type of tooth attachment to the underlaying bone, or by the ability to replace the teeth.<br>Findings: Here, we provide full datasets of microtomography scans and 3D models of reptilian dentitions and skulls. We selected representative species for each of nine reptilian families, based on their characteristic dental features. As there are at least four different types of tooth-bone attachments, ranging from mammalian-like thecodont attachment found in crocodilian to the simple acrodont implantation observed in some lizards, we aimed to evaluate species with different types of tooth-bone attachments. Moreover, another interesting feature varying in reptilian species is the complexity of tooth shape or the number of tooth generations, which can be associated with the type of tooth attachment to the jawbone. Therefore, selected model species also include animals with distinct tooth morphology along the jaw or different number of tooth generations. Development of tooth attachment and relationship of tooth to the jaw can be further analyzed in detail on large collection of pre-hatching stages of chameleon. Next, we introduce different possibilities how these datasets can be further used to study tooth-bone relationship or tooth morphology in 3D space. Moreover, these datasets can be valuable for additional morphological and morphometric analyses of reptilian skulls or their individually segmented skeletal elements.<br>Conclusions: Our collection of micro-computed tomography scans can bring new insight into dental or skeletal research. Broad selection of reptilian species together with their unique dental features and high quality of these scans including complete series of developmental stages of our model species, provide large opportunities of their biological reusage. Scans can be further used for virtual reality, 3D printing, or follow up usage of these models in education.
**Corresponding Author:**	Marcela Buchtova<br>Institute of Animal Physiology and Genetics CAS: Ustav zivocisne fyziologie a genetiky Akademie ved Ceske republiky<br>Brno, CZECH REPUBLIC
**Corresponding Author Secondary Information:**	
**Corresponding Author's Institution:**	Institute of Animal Physiology and Genetics CAS: Ustav zivocisne fyziologie a genetiky Akademie ved Ceske republiky
**Corresponding Author's Secondary Institution:**	
**First Author:**	Michaela Kavkova
**First Author Secondary Information:**	
**Order of Authors:**	Michaela Kavkova
	Marie Šulcová
	Tomáš Zikmund

Powered by Editorial Manager® and ProduXion Manager® from Aries Systems Corporation

	Martin Pyszko
	Jozef Kaiser
	Marcela Buchtova
**Order of Authors Secondary Information:**	
**Response to Reviewers:**	Editor:<br>In addition, it was brought to my attention that the following two relevant recent publications should be cited and briefly discussed in your data note:<br><br>Watanabe et al. 2019, PNAS<br>Ecomorphological diversification in squamates from conserved pattern of cranial integration<br><br>Lafuma et al. 2021, Nature Communications<br>Multiple evolutionary origins and losses of tooth complexity in squamates<br><br>Please also ensure that your revised manuscript conforms to the journal style, which can be found in the Instructions for Authors on the journal homepage. In particular, please structure your abstract ("background, findings, conclusions") and use numbered references.<br>ANSWER:<br>In revised version, we included recommended references in the text of manuscript as well as reference list.<br>Abstract was adjusted to journal format.<br>References were switched to numbered format.<br><br>Reviewer 1<br>Reviewer #1: This data note presents microCT scans of a variety of reptiles focusing on tooth morphology. It is very interesting and well presented, with nice images and high quality data. A note on the data - the image stacks I viewed are good quality but the STL's I checked all have something wrong, I could not open these, you will have to check these. The acquisition is well reported and I think the work can be published. I think the authors might find it interesting that previous work in Gigascience also presented microCT scans of a range of venomous snake fangs (but not the gaboon viper), it might be interesting to reference this<br>https://doi.org/10.1093/gigascience/gix126<br><br>ANSWER:<br>Thank you for your kind comments.<br>We hope STL´s are working now, we also tried to open them from several computers, we have not notice further difficulties. But it is possible that there can be some troubles while using computer with lower memories as we found this as limitation of usage. In case of any further difficulties please could you contact Michaela Kavkova (Michaela.Kavkova@ceitec.vutbr.cz) to find where these difficulties arise.<br>Mentioned reference is now included in the text as well as reference list.<br><br>Reviewer 2<br>* Timon lepidus (files are named Lacerta lepida)<br>ANSWER:<br>In the text, we are now uniformly call this species as Timon lepidus, but we also included older still commonly used name Lacerta lepida in the manuscript. The database did not allow us to rename files to Timon lepidus but we can do loading of these files again in case reviewer and editor would prefer it.<br><br>Minor comment 1<br>According to Table 3, the Stage 1 Chamaeleo calyptratus embryo is older than the Stage 2 Chamaeleo calyptratus embryo. This would suggest that staging is based on alternative criteria than age, such as the weight of the embryo. Can the authors please clarify what criteria were used to stage the chameleon embryos?<br>ANSWER:<br>Our staging system is based both on the age of the embryos and also the weight of

	each individual. By such, we can easily compare also the animals from different clutches since they can develop in different speed. This was also the case of chameleons named stage 1 and chameleon stage 2 where the animal was younger, but the embryo was heavier (0.37 g) and more developed. The animal came from different clutch in comparison to stage 1 chameleon with the weight of only 0.25 g and it was less developed. In addition to length of pre-hatching development and their weight which is more corresponding to actual developmental stage, we also evaluate morphological features, which help us to better determine the level of development in oviparous reptiles where the length of development varies between clutches as well as inside individual clutch.<br><br>Minor comment 2<br>For the Stage 6 Chamaeleo calyptratus embryo, a 3D surface reconstruction (STL format) is provided of segmented dentition. Can the authors please clarify whether they will additionally be submitting a 3D surface reconstruction (STL format) of Stage 6 embryo craniofacial skeletal components to the GigaScience Database?<br>ANSWER:<br>Unfortunately, we do not have available scans of the upper jaw. For this larger animal, we wanted to have similar resolution scans to the smallest animals therefore we had to perform scanning of just proportion of head. We are aware this weakness and impossibility to analyze upper jaw, but we hoped that readers can use at least lower jaw scans for teeth and certain skeletal analyses.<br><br>Thank you for your kind comments.
**Additional Information:**	
**Question**	**Response**
Are you submitting this manuscript to a special series or article collection?	No
**Experimental design and statistics**<br><br>Full details of the experimental design and statistical methods used should be given in the Methods section, as detailed in our Minimum Standards Reporting Checklist. Information essential to interpreting the data presented should be made available in the figure legends.<br><br>Have you included all the information requested in your manuscript?	No
If not, please give reasons for any omissions below.<br><br>as follow-up to "**Experimental design and statistics**<br><br>Full details of the experimental design and statistical methods used should be given in the Methods section, as detailed in our Minimum Standards Reporting Checklist.	There is not statistic used in recent data set.

Information essential to interpreting the data presented should be made available in the figure legends.<br><br>Have you included all the information requested in your manuscript?<br><br>"	
**Resources**<br><br>A description of all resources used, including antibodies, cell lines, animals and software tools, with enough information to allow them to be uniquely identified, should be included in the Methods section. Authors are strongly encouraged to cite Research Resource Identifiers (RRIDs) for antibodies, model organisms and tools, where possible.<br><br>Have you included the information requested as detailed in our Minimum Standards Reporting Checklist?	Yes
**Availability of data and materials**<br><br>All datasets and code on which the conclusions of the paper rely must be either included in your submission or deposited in publicly available repositories (where available and ethically appropriate), referencing such data using a unique identifier in the references and in the "Availability of Data and Materials" section of your manuscript.<br><br>Have you have met the above requirement as detailed in our Minimum Standards Reporting Checklist?	Yes

Manuscript

Click here to access/download;Manuscript;GigaScience Reptiles R1 19.12.21.doc

DATA NOTE

X-ray microtomography imaging of craniofacial hard tissues in selected reptile species with different types of dentition

Michaela Kavková[1], Marie Šulcová[2,3], Tomáš Zikmund[1], Martin Pyszko[4], Jozef Kaiser[1] and Marcela Buchtová[2,3]

[1] Central European Institute of Technology, Brno University of Technology, Brno, Czech Republic

[2] Department of Experimental Biology, Faculty of Science, Masaryk University, Brno, Czech Republic

[3] Laboratory of Molecular Morphogenesis, Institute of Animal Physiology and Genetics, v.v.i., Czech Academy of Sciences, Brno, Czech Republic

[4] Department of Anatomy, Histology and Embryology, Faculty of Veterinary Medicine, University of Veterinary Sciences Brno, Brno, Czech Republic

Author for correspondence:

Dr. Marcela Buchtova

Laboratory of Molecular Morphogenesis

Institute of Animal Physiology and Genetics, v.v.i.

Czech Academy of Sciences

Veveri 97, 602 00 Brno,

Czech Republic

Email: buchtova@iach.cz,

Abstract

Background: Reptiles exhibit a large heterogeneity in the teeth morphology. The main variability is comprised by the different tooth shape, the type of tooth attachment to the underlaying bone, or by the ability to replace the teeth.

Findings: Here, we provide full datasets of microtomography scans and 3D models of reptilian dentitions and skulls. We selected representative species for each of nine reptilian families, based on their characteristic dental features. As there are at least four different types of tooth-bone attachments, ranging from mammalian-like thecodont attachment found in crocodilian to the simple acrodont implantation observed in some lizards, we aimed to evaluate species with different types of tooth-bone attachments. Moreover, another interesting feature varying in reptilian species is the complexity of tooth shape or the number of tooth generations, which can be associated with the type of tooth attachment to the jawbone. Therefore, selected model species also include animals with distinct tooth morphology along the jaw or different number of tooth generations. Development of tooth attachment and relationship of tooth to the jaw can be further analyzed in detail on large collection of pre-hatching stages of chameleon. Next, we introduce different possibilities how these datasets can be further used to study tooth-bone relationship or tooth morphology in 3D space. Moreover, these datasets can be valuable for additional morphological and morphometric analyses of reptilian skulls or their individually segmented skeletal elements.

Conclusions: Our collection of micro-computed tomography scans can bring new insight into dental or skeletal research. Broad selection of reptilian species together with their unique dental features and high quality of these scans including complete series of developmental stages of our model species, provide large opportunities of their biological reusage. Scans can be further used for virtual reality, 3D printing, or follow up usage of these models in education.

Keywords: micro-CT, 3D imaging, reptiles, tooth-bone attachment, skull, craniofacial bones, tooth replacement

Background

Teeth are composed from the hardest tissues in the body of all living and extinct animals. This tissue is resistant against external intervention; therefore, its structure is sometimes the only entity persisting for ages to be useful for palaeontologists to describe each fossil and the placement of samples in the context of other extinct animals. This organ exhibits an extensive heterogeneity reflecting animal lifestyle. In herbivores, the structure of skull including teeth is adapted to improve the grinding of the plant food. In contrast, the shape of teeth in carnivorous predators is designed for the capture and processing of the prey's flesh [1].

Large heterogeneity in tooth shapes and more importantly, the way the dentition is placed in the jaw, was found especially in reptiles [2,3]. There are at least four distinct types of tooth-bone attachments, ranging from mammalian-like thecodont implantation, observed in crocodiles, to simpler acrodont type found in some lizards [4]. Tooth-bone implantation can also exhibit region-specific character across the jaw. In bearded dragons (*Pogona vitticeps*), two types (acrodont and pleurodont) of tooth-bone attachments are present within the jaw with the pleurodont type located in the rostral area and the acrodont in the caudal part of the jaw [5]. Specialised complex tissue serving as an adhesive component connecting the jawbone and teeth in monitor lizards was called as plicidentin [6] (Maxwell et al., 2011).

The way the tooth is placed to the jawbone also reflects the number of tooth generations, which can be initiated in certain species. Tooth replacement represents another interesting feature varying in reptiles. In chameleons, which are strictly monophydont with only one tooth generation, teeth are firmly attached to the jawbone, and if the tooth is lost due

to injury or during a fight, the jawbone is extensively damaged [7]. Also, the whole process of odontogenesis across individual developmental stages is peculiar, especially because of the asymmetrically developing tooth germ ending up with the symmetrically ankylosed teeth [8]. However, most of the reptiles (e.g., geckos, anolis, skinks, ameiva, iguanas, or snakes) possess an unlimited and life-long supply of new tooth generations. The type of tooth attachment is less robust in these species to ensure the constant loss of functional teeth and growth of the replacement teeth [2,9-12].

Here, we provide full datasets of our micro-computed tomography (micro-CT) scans from various reptilian species, which can be further used for comparative morphology of the different types of tooth-bone attachments in selected representants encompassing key species with distinct dental features. The provided datasets are also suitable for the evaluation of tooth shape differences between species or across the jaws, dental replacement patterns, to determine the facial and cranial bone structure in these species, or to analyse a large spectrum of morphometric parameters in 2D or 3D across reptiles. Moreover, the offered datasets from the broad spectra of chameleon embryos of different age enlarged amount of data available for further analysis such as the progression of reptilian odontogenesis with emphasis on changes in morphology of the teeth and skeletal elements with the possibility to evaluate alteration in their relationship in time.

Sampling strategy

For this study, we enlarged the list of scanned model species, which have been used for the purpose of our previously published studies focused on tooth development [7,8,13,14] by the species possessing further unique features among reptiles. As a source of information for the selection of key reptilian specimens, we used a detailed review discussing the different aspects of the tooth to bone relationship across Amniotes [2]. Therefore, different animal

species were selected according to their unique type of tooth shape and tooth-bone relationship on which we are preferentially focused on in our studies; however, these datasets can be used for numerous additional analyses that we also shortly introduce here.

Anolis equestris is a representative of Iguanidae, which possess so-called Iguana-type implantation. In this type of attachment, the labial side of the tooth is attached to the high labial wall of the jaw, therefore it remains shorter. However, the lingual side of the tooth is extending deeper into the jaw to contact the jawbone, which makes this type of the attachment strongly asymmetrical. According to the replacement potential, this species is pleurodont with an unlimited number of tooth generations [11]. The shape of their teeth varies along the jaw. Rostral teeth are simple and conical and do not exhibit any morphological uniqueness. However, in the caudal area of the jaw, the tip of the teeth is larger and split into three cusps: one central and two lateral cusps.

Another selected species, commonly demonstrated as a typical pleurodont and polyphyodont, is *Paroedura picta*, belonging to the family of Gekkonidae. Geckos possess uniformly shaped teeth along the whole jaw. On the top of each tooth, there is deep groove, which divides the tooth tip into two ridges along the whole tooth.

Pleurodont dentition with homogenous tooth shape is typical for *Scincus scincus* from the Scincidae family. Similar to the gecko, there is an apparent uniformity in their tooth shape along the jaw. Teeth are sharp with the visible deep dent at their tip. There are several small teeth located on the palate. The whole skull is covered by scales, forming an armour-like structure, which had to be segmented to visualise the bones of the skull and teeth before 3D processing of micro-CT images.

Timon lepidus (previously called *Lacerta lepida*, Lacertidae), a lizard species commonly found in southwestern Europe, is another representative of pleurodont species. In the rostral part of the jaw, teeth exhibit simple monocuspid morphology, while in the caudal

jaw area, teeth display three cusps on their tip similarly to the above-mentioned lizard species. There are also distinct palatal teeth attached to the pterygoid bone.

As a transitional species bearing both pleurodont and acrodont type of the dentition, we selected *Pogona vitticeps* (Agamidae). Bearded dragons possess pleurodont teeth in the most rostral part of the jaw, which exhibit the ability of life-long replacement. However, most of the teeth are acrodontly ankylosed to the underlying jawbone [5]. These two types of dentitions are easily recognisable since the pleurodont teeth are monocuspid and sharply point to each other. In contrast, the acrodont dentition forms together with the jawbone compact structure, where caudal teeth are closely attached to each other on their lateral sides with complex shape consisting of one central and two lateral cusps.

Completely ankylosed teeth are found in *Chamaeleo calyptratus* (Chamaeleonidae). There is visible heterogeneity in tooth shape along the jaw, ranging from smaller teeth in the very rostral part of the jaw and tricuspid teeth in the caudal area, where teeth are also noticeably larger. The detailed description of chameleon dentition has been previously introduced in a number of publications, where the micro-CT was used as one of the key methods [7,8,14]. In these studies, mostly the embryos were used to describe the process of odontogenesis. Therefore, so far, we know that the chameleon teeth germs are developing asymmetrically, closely reminding the pleurodont implantation [8]. Also, the patterning of the tooth germ initiation is precisely coordinated, with first calcified tooth appearing caudally followed by next teeth placed rostrally [15].

A unique type of tooth-jaw attachment was found in monitor lizards, where the infoldings of plicidentin contribute to the junction between the tooth and the jaw [6]. To examine this so-called Varanus-type of implantation, we performed micro-CT scans of *Varanus beccarii* (Varanidae). The overall morphology of individual teeth in Varanus is rather simple and successional tooth generations grow in posterior-lingual direction to the functional teeth [11].

In *Salvator rufescens* (Teiidae), the heterogeneity of dentition includes both tooth shape and the type of tooth to bone attachment. In the rostral area, simple and sharp monocuspid teeth are located. More caudally, the simplicity of the tooth is replaced by teeth with bulbous shape, which helps with crunching the prey such as shells, small birds, or rodents. This type of implantation, often termed as Dracaena-type, is unique since the teeth are sat in shallow sockets, which are deeper in the rostral area [11].

Bitis gabonica is a representative of poisonous snakes (Viperidae) with venomous teeth located in the rostral area of the upper jaw. Their venomous teeth represent the longest fangs of venomous snakes with a size of about 5 cm in length. Gaboon vipers also possess simple conical teeth arranged into two rows in the upper jaw (inner - palatal and outer - maxillary row) and one row (mandibular) in the lower jaw. Teeth, including fangs, are replaced several times through life (polyphyodont dentition) and they exhibit typical pleurodont type of attachment [16,17].

As an example of nonvenomous snakes, we selected *Python regius* (Pythonidae), which is a constrictor lacking typical venomous teeth. Simple shaped teeth with sharp monocuspid morphology are curved caudally deeper to the python jaw; similar to vipers, they are arranged into two rows in the upper jaw and one row in the lower jaw. Their polyphyodont dentition is associated with pleurodont type of tooth attachment along whole jaws [10,16,17].

Besides the squamates species, we also examined a specimen of *Caiman crocodilus* (Alligatoridae). All Crocodilians are known to possess tooth implantation resembling those in mammals, called thecodont gomphosis. In this type of dentition, teeth are situated in deep bony alveolus with the presence of bundles of both mineralised and non-mineralised periodontal ligaments mediating the junction force between the tooth and jawbone [18].

Source of samples

All analysed specimens originated from private breeders. Deceased animals were part of the collection of the Department of Anatomy, Histology and Embryology at the Faculty of Veterinary Medicine, University of Veterinary Sciences Brno (Brno, Czech Republic).

All embryonic stages of chameleon were obtained from a commercial breeder. Embryos were collected at six different developmental stages and fixed in 4% PFA at least overnight. All manipulations followed the specific rules for working with alive embryos as specified by the Central Commission for Animal Welfare of Ministry of Agriculture of the Czech Republic (§16a law No. 246/1992 Sb., for animal protection against cruelty). All analyses were performed in accordance with the guidelines, regulations and experimental protocols approved by the institutional and licensing committee including rules run by the Laboratory Animal Science Committee of the IAPG, v.v.i. (Liběchov, Czech Republic). No experiments were performed on live embryos.

Micro-CT scanning

Frozen reptile skulls were thawed in absolute ethanol. To stabilize the motions during the micro-CT scanning, all of the samples were located into the plastic container selected to fit the sample and embedded in 1% agarose gel.

Eleven samples of various reptilian adult heads and six samples of chameleon embryo stages were selected for the detailed micro-CT analysis of hard tissue morphology as described higher (**Fig. 1, Table 1**).

The individual samples of chameleon embryos were selected to showcase the development of the ankylotic teeth in pre-hatching stages, the list of the samples is enrolled in **Table 3**. The micro-CT scanning was performed using laboratory system GE phoenix v|tome|x L 240 (GE Sensing & Inspection Technologies GmbH, Germany), equipped with a

180 kV/15W maximum power nanofocus X-ray tube and high contrast flat panel detector dynamic 41|100 (number of pixels: 4048 x 4048 px, pixel size 100 μm). The measurements were carried out in the air-conditioned cabinet (21°C). Instrumental settings for each sample are displayed in **Table 1** and **Table 4**. The tomographic reconstruction was performed using the software GE phoenix datos|x 2.0 (GE Sensing & Inspection Technologies GmbH, Germany).

Data quality control and limitations

Since all analyzed samples of adult reptile skulls were preserved prior to scan and then they were handled in the same way, the main variable in the quality of data was generated by the different voxel size of each dataset. The above-mentioned variability was caused by the difference in sizes through the samples. Considering the whole set of samples, the smallest sample was the skull of *Scincus scincus* – 21 mm with voxel size of 13 µm and the largest skull was *Salvator rufescens* – 98 mm with voxel size of 48 µm.

The difference in voxel size was directly linked to dimensions of the sample. Considering the field of view given by the utilized detector and the cone beam geometry of the X-ray source of GE L240, this fact concludes in simple rule: the smaller the sample is, the closer to the x-ray source in the cone beam it can be placed, which finally results in smaller voxel size (higher resolution). Even though the samples display variable voxel size, this does not necessarily have to limit the analysis of the data. Larger specimens demonstrate the voxel size with lower resolution than the smaller samples, however analysed structures are larger in these animals, therefore they are easily recognizable.

In case of the chameleon embryo samples the dimensions of embryo were mostly the same and the difference in voxel size in smallest embryo to the largest one was only 1 µm.

Samples of all reptile skulls and chameleon embryos were scanned in intact form (the 90% ethanol was used to fix the tissues in the adult reptile skulls and the 4% PFA was used to fix the embryonic samples). Based on the principle of micro-CT imaging of the samples in native form without any type of staining, only the dense mineralized structures such as tooth and bones (and in case of *Scincus scincus* the scales) can be visualized. Therefore, one of the limitations of the presented dataset is omitted information about the soft tissue appearance and morphology in adult tissues. As our main aim was to examine reptile skulls and tooth to bone attachment, the staining agent would affect visualization of smaller bone borders, which would become poorly distinguishable from background after counterstaining.

Tooth spacing analyses by polyline tool

Utilization of polyline tool was used to create an open “unrolled” images of the lower jaw (**Fig. 2A**). Each dataset was oriented based on the main anatomical directions. In the coronal and horizontal direction, the sections were oriented to be symmetrically aligned. In the sagittal plane, where the symmetry is not present, the sections were rotated to visualize as many teeth as was possible for the lower jaw. In oriented dataset, points defining the polyline were placed on the selected tips of the teeth in the lower jaw (**Fig. 2B**). Finally, the unrolled images of the lower jaw were generated in new plane defined by the polyline. The unrolled images can be used to evaluate replacement patterns (**Fig. 2**) or to display and determine changes in teeth spacing throughout the lower jaw as visualized in embryonic chameleon stages (**Fig. 3**).

The analysis of the teeth spacing in the “unrolled” data was slightly complicated by the fact that during the initial freezing of samples, some of them were not frozen in optimal horizontal position and after the thawing, some structures such as loose joint in the rostral part of jaw in snakes, were slightly bended resulting in not perfectly oriented and unsymmetrical

images. Even if in certain samples the symmetry of the “unrolled” images was not ideal, required information about the teeth spacing can be extracted from two separate images for each half of the jaw.

Micro-CT data analysis of tooth-bone attachment

Morphology of tooth-bone interface and their regional differences were evaluated from micro-CT scans in VGStudio MAX 3.3 software (Volume Graphics GmbH, Germany). To achieve the optimal transversal sections through the jaw, previously generated polyline was used. The transversal sections were produced in perpendicular plane to the original horizontal section. The coronal sections were oriented based on the curve of the polyline (**Fig. 4**).

Such projections could be further used for the description of different tooth-bone relationship with regard to the individual bone appearance and the nature of tissue mediating the interface. Moreover, since the section could be placed anywhere throughout the skull, such structures as palatal teeth appearance and their distribution, temporo-mandibular joint morphology or spatial relationship among individual craniofacial bones can be studied.

Wall thickness analysis of skulls

MicroCT scans can be used to evaluate the hard tissue density and thickness, which could serve as a source of valuable information for deciphering different aspects of skull anatomy as well as their interspecies differences.

VG Studio software enable two methods of wall thickness analysis: ray-based method and sphere-based method. Ray based method defines the wall thickness by searching the opposite surface by sending a measurement ray perpendicularly to the current surface. Calculated wall thickness is then defined by the shortest distance between two crossed rays

from opposite surfaces. The sphere-based method evaluates the wall thickness by fitting spheres inside the sample in 3D space. The thickness of the analyzed structure is then defined by the diameter of the fitted sphere.

In case of complex bone structure, the sphere-based measurement of wall thickness analysis is more accurate, therefore here we introduce data from this type of analyses on our selected species (**Fig. 5, Video 1**). Color scale of the wall thickness analysis displays the hard tissues elements, which exhibits more gracile morphology, and we were able to distinguish them from reinforced elements. This information can be beneficial for the estimation of strains influencing specific parts of the skull leading to food processing adaptations.

Analysis of single tooth morphology

The high resolution of obtained micro-CT data enables precise segmentation of specific structures of the analyzed sample. As an example, the teeth of several species were segmented. The process of segmentation includes the global thresholding step to define the hard tissues from background. In the following step, the object of interest (in our case the venomous tooth of *Bitis gabonica* and replacement tooth of *Caiman crocodilus*) is roughly marked by 3D brush, by intersecting the created rough model of tooth with the defined bones, the precise model of tooth is created.

Samples of *Bitis gabonica* and *Caiman crocodilus* were selected to display some possibilities for more detailed tooth analysis. These species represent different types of tooth morphology (venomous teeth in *Bitis gabonica* and simple conical tooth in *Caiman crocodilus*) as well as tooth implantation (pleurodont implantation in *Bitis gabonica* and thecodont implantation in *Caiman crocodilus*).

In case of *Bitis gabonica*, venom teeth from the left side of the skull were segmented and visualized (**Fig. 6A-D, Video 2**). The volume of teeth can be easily measured (the volume

of the largest visualized tooth was 3.88 mm^3 in this sample) (**Fig. 6**). Transparent visualization enables us to determine dental pulp morphology, analyze its shape in individual teeth or its communication to bone marrow as we discussed previously in chameleon [7].

Micro-CT analysis of *Caiman crocodilus* teeth enabled us to visualize next generation of teeth hidden in the currently employed tooth (**Fig. 7, Video 3**). The shape and volume of individual teeth can be evaluated along the jaw and compared across regions. Here, the smaller tooth volume was 1.22 mm^3 and the volume of the largest tooth was 11.44 mm^3.

This type of analysis offers great potential for investigation number of aspects such as precise tooth shape and morphology description, dental pulp filling and/or venomous canal formation, replacement pattern in polyphyodont species and visualization of individual tooth generation. All of the abovementioned analyses can be applied to any craniofacial structure of interest. The only condition is previous careful segmentation of analyzed structure before their further analyses.

Palatal teeth analyses

Presence of palatal teeth in reptiles is diverse (**Fig. 8**). Some species does not bare teeth in the palatal area (chameleons), while in other species, their play important role in food transport through the oral cavity (venomous snakes) [19]. Palatal teeth can also serve as a tool for processing of plant food or cracking the hard-shell prey [20]. The overall morphology of palatal teeth is rather simple in comparison to the marginal teeth [19]. The presence or absence of structures such as palatal teeth could also predicate evolutionary similarity across the reptiles. Therefore, offered dataset could broaden this under evaluated topic and offer the valuable information for further study.

Segmentation of selected craniofacial bones

Large potential of our dataset is also in the possibility to evaluate not only the dentition morphology or individual teeth but also other hard craniofacial tissues. Thus, dataset represents robust system, which could be applied for any of scanned skeletal structure examination. The necessity of precise segmentation is here immense, because once the studied structure is visibly discriminated, the further evaluation of its morphology and/or the interspecies differences is much easier and more accurate. However, this tool brings another possibility how to describe each skeletal structure in detail separately or in context to individual surrounding elements. Besides the study of interspecies differences, segmented structures could be used for study of intersex variability in skull morphology or dynamics of bone development/progress.

Here, VG Studio Max software was employed to segment the craniofacial bones (**Fig. 9, 10, Video 4**). The first used module was "surface determination", which was applied to distinguish the bones from the background. The region of interest (ROI) of all bones was determined. In the following step, the tool "3D brush" was used to mark the approximate ROI of selected bones contributing to the palate. The final step was then generation of the ROI containing only the selected bone by intersecting the ROI of all bones with the marked ROI of the bone.

Volume analyses of skulls and skeletal elements

To make our analysis of segmented skull bones more detailed and therefore highly applicable, the volumes analysis was subsequently applied. Volumes of whole skull or bones of interest can be analyzed from 3D data (see examples in **Table 2**). Acquired data could be used for assessing of interspecies differences in bone volumes, which could be then used for deciphering of evolutionary questions and other related topics.

Further biological potential

Indisputable biological potential of presented dataset lies in the number of different species which were scanned, quality of scans as well as large possibilities for subsequent analysis on acquired data. Therefore, presented dataset can be used for further studies concerning comparative ECO-EVO or morphological/anatomical topics. Here, we introduce different possibilities of data re-use potential with the main focus on craniofacial structures.

The acquired data could be helpful in the study of different types of tooth-bone attachment and implantations, which is, according to recent publications broadly studied topic [8,10,11,21-24] and varies across the species. Another interesting feature concerning the teeth is the analysis of tooth spacing to which we used specific tool in VG studio Max allowing to "unroll" the jaw for displaying all the teeth at once. The micro-CT analysis also enables to precisely describe the process of tooth replacement with closer focus on the position of the next tooth generations in comparison to the first generation or deciphering of general replacement pattern, which can be also evaluated in our dataset. Thanks to the segmentation tool, study of the poison flow throughout the venomous canal in fangs is possible or determination of dental pulp changes across the jaw.

Moreover, the obtained data could be used for further studies by those who are particularly interested in reptilian skull anatomy (e.g. temporomandibular joint, columella, palatal bones or other structures) and its evolutionary differences across diverse species. These skeletal elements can be evaluated by the tool which enable the precise segmentation of individual structure. These segmented bones could be further processed to get the precise information about their shape, volume and relationship to other surrounding structures in 3D. Furthermore, wall thickness analysis can be useful to study structural aspects of skeletal elements.

To broaden the knowledge about the reptilian skull anatomy, morphology and development, the scans of multiple chameleon embryos are enclosed. By combination of all introduced analysis, extremely comprehensive and detailed information about both dental and bone tissue morphology in selected model species across reptilians will be gain.

Popularization/educational potential of presented dataset

The 3D printed models of the whole analysed reptilian skulls or individual segmented skeletal elements of interest can be also used as an innovative learning support for the university or secondary school students. Both 3D printed and virtual reality models could be also presented to general public, e.g. museums without any need to possess original skulls or taxidermy mounts (**Fig. 11**). Usage of 3D scans for virtual reality enable not only detailed view on individual structures, however, also walking through skull or inside of individual skeletal elements.

Data Availability

Volumetric data of all scanned animals are available in 8 bit tiff stack files. These tiff stacks include the raw tomographic sections. The data were reduced from original 16 bit to 8 bit to make the data more compact to download and to make it more available for analysis, considering the larger datasets needs high computational power to even open them. The stl models of skulls were created for all analyzed samples, these models are highly detailed which makes them suitable for 3D print in higher magnifications. Additional tiff stacks of demonstrated analyses (unrolled lower jaw) are included. Finally, the videos displaying in detail individual analyses (such as wall thickness analyses, teeth or bone segmentation) or transversal sections through the head are included.

Competing interests

The author(s) declare that they have no competing interests.

Funding

This study was supported by the Ministry of Health, Czech Republic (AZV/NU20-06-00189/2020).

Authors' contributions

Data analyses: MK

Visualization: MK, MS

Validation: TZ

Conceived and designed experiments: TZ, MB

Grant support: MB

Contributed reagents and materials: MP, JK

Software: TZ, JK

Writing of original manuscript: MK, MS, MB

Writing - review & editing: TZ, MP, MB

Acknowledgement:

Authors would like to thanks to Oldřich Zahradniček for samples of *Paroedura picta* and *Pogona vitticeps.*

References

1. Watanabe A, Fabre AC, Felice RN, Maisano JA, Müller J, Herrel A, Goswami A 2019. Ecomorphological diversification in squamates from conserved pattern of cranial integration. Proc Natl Acad Sci USA 116(29):14688-14697.

2. Bertin, T.J.C., Thivichon-Prince, B., LeBlanc, A.R.H., Caldwell, M.W., Viriot, L., 2018. Current Perspectives on Tooth Implantation, Attachment, and Replacement in Amniota. Frontiers in Physiology 9, 20.

3. Lafuma F, Corfe IJ, Clavel J, Di-Poï N, 2021. Multiple evolutionary origins and losses of tooth complexity in squamates. Nat Commun 12(1):6001.

4. Gaengler, P., 2000. Evolution of tooth attachment in lower vertebrates to tetrapods. Cambridge University Press, Cambridge, pp. 173–185.

5. Salomies, L., Eymann, J., Khan, I., Di-Poï, N., 2019. The alternative regenerative strategy of bearded dragon unveils the key processes underlying vertebrate tooth renewal. Elife 8.

6. Maxwell, E.E., Caldwell, M.W., Lamoureux, D.O., Budney, L.A., 2011. Histology of Tooth Attachment Tissues and Plicidentine in Varanus (Reptilia: Squamata), and a Discussion of the Evolution of Amniote Tooth Attachment. Journal of Morphology 272, 1170-1181.

7. Dosedelova, H., Stepankova, K., Zikmund, T., Lesot, H., Kaiser, J., Novotny, K., Stembirek, J., Knotek, Z., Zahradnicek, O., Buchtova, M., 2016. Age-related changes in the tooth-bone interface area of acrodont dentition in the chameleon. Journal of Anatomy 229, 356-368.

8. Kavkova, M., Sulcova, M., Dumkova, J., Zahradnicek, O., Kaiser, J., Tucker, A.S., Zikmund, T., Buchtova, M., 2020. Coordinated labio-lingual asymmetries in dental and bone development create a symmetrical acrodont dentition. Scientific Reports 10, 16.

9. Delgado, S., Davit-Beal, T., Allizard, F., Sire, J.Y., 2005. Tooth development in a scincid lizard, Chalcides viridanus (Squamata), with particular attention to enamel formation. Cell and Tissue Research 319, 71-89.

10. du Plessis, A., Broeckhoven, C., le Roux, S.G., 2017. Snake fangs: 3D morphological and mechanical analysis by microCT, simulation, and physical compression testing. Gigascience 7.

11. LeBlanc, A.R.H., Paparella, I., Lamoureux, D.O., Doschak, M.R., Caldwell, M.W., 2021. Tooth attachment and pleurodont implantation in lizards: Histology, development, and evolution. Journal of Anatomy 238, 1156-1178.

12. Zahradnicek, O., Horacek, I., Tucker, A.S., 2012. Tooth development in a model reptile: functional and null generation teeth in the gecko Paroedura picta. Journal of Anatomy 221, 195-208.

13. Hampl, M., Dumkova, J., Kavkova, M., Dosedelova, H., Bryjova, A., Zahradnicek, O., Pyszko, M., Macholan, M., Zikmund, T., Kaiser, J., Buchtova, M., 2020. Polarized Sonic Hedgehog Protein Localization and a Shift in the Expression of Region-Specific Molecules Is Associated With the Secondary Palate Development in the Veiled Chameleon. Frontiers in Cell and Developmental Biology 8, 29.

14. Sulcova, M.L., Zahradnicek, O., Dumkova, J., Dosedelova, H., Krivanek, J., Hampl, M., Kavkova, M., Zikmund, T., Gregorovicova, M., Sedmera, D., Kaiser, J., Tucker, A.S., Buchtova, M., 2020. Developmental mechanisms driving complex tooth shape in reptiles. Developmental Dynamics 249, 441-464.

15. Buchtova, M., Zahradnicek, O., Balkova, S., Tucker, A.S., 2013. Odontogenesis in the Veiled Chameleon (Chamaeleo calyptratus). Archives of Oral Biology 58, 118-133.

16. LeBlanc, A.R.H., Lamoureux, D.O., Caldwell, M.W., 2017. Mosasaurs and snakes have a periodontal ligament: timing and extent of calcification, not tissue complexity, determines tooth attachment mode inreptiles. Journal of Anatomy 231, 869-885.

17. Zaher, H., Rieppel, O., 1999. Tooth implantation and replacement in squamates, with special reference to mosasaur lizards and snakes. American Musem Novitates, 3271:1-19

18. McIntosh, J.E., Anderton, X., Flores-De-Jacoby, L., Carlson, D.S., Shuler, C.F., Diekwisch, T.G.H., 2002. Caiman periodontium as an intermediate between basal vertebrate ankylosis-type attachment and mammalian "true" periodontium. Microscopy Research and Technique 59, 449-459.

19. Mahler, L., Kearney, M., 2005. The palatal dentition in squamate reptiles: Morphology, development attachment, and replacement. Integrative and Comparative Biology 45, 1036-1036.

20. Matsumoto, R., Evans, S.E., 2017. The palatal dentition of tetrapods and its functional significance. Journal of Anatomy 230, 47-65.

21. Haridy, Y., LeBlanc, A.R.H., Reisz, R.R., 2018. The Permian reptile Opisthodontosaurus carrolli: a model for acrodont tooth replacement and dental ontogeny. J Anat 232, 371-382.

22. LeBlanc, A.R.H., Brink, K.S., Cullen, T.M., Reisz, R.R., 2017. Evolutionary implications of tooth attachment versus tooth implantation: a case study using dinosaur, crocodilian, and mammal teeth. Journal of Vertebrate Paleontology 37, 19.

23. LeBlanc, A.R.H., Brink, K.S., Whitney, M.R., Abdala, F., Reisz, R.R., 2018. Dental ontogeny in extinct synapsids reveals a complex evolutionary history of the mammalian tooth attachment system. Proceedings of the Royal Society B-Biological Sciences 285(1890):20181792.

24. Palci, A., LeBlanc, A.R.H., Panagiotopoulou, O., Cleuren, S.G.C., Abraha, H.M., Hutchinson, M.N., Evans, A.R., Caldwell, M.W., Lee, M.S.Y., 2021. Plicidentine and the repeated origins of snake venom fangs. Proceedings of the Royal Society B-Biological Sciences 288(1956):20211391.

FIGURE LEGENDS:

Figure 1: Skull morphology of analyzed reptiles (lateral view of micro-CT images).

A) *Bitis gabonica*, B) *Anolis equestris*, C) *Caiman crocodilus*, D) *Chamaeleo calyptratus*, E) *Timon lepidus*, F) *Paroedura picta*, G) *Pogona vitticeps*, H) *Python regius*, I) *Salvator rufescens*, J) *Scincus scincus*, K) *Varanus beccarii*, L-Q) six pre-hatching stages of *Chamaeleo calyptratus*.

Figure 2: Visualization of unrolled lower jaw using polyline tool.

Examples of *Paroedura picta* and *Varanus beccarii* skulls, the design of polyline and unrolling of the lower jaw is displayed. In the 3D render image, the points defining the polyline are presented as red dots and produced polyline is labelled in green color.

Figure 3: Visualization of tooth spacing while using unrolled data of chameleon embryonic jaw.

The exact placement of the unrolled section through the lower jaw is marked by the purple plane shown in 3D render. In the unrolled image the distances between individual teeth are measured.

Figure 4: Tooth-bone attachment in different reptiles displayed on transversal sections through the jaw.

Orange plane in the 3D render of skulls for each animal demonstrate the positioning of section through the jaw.

A) In *Timon lepidus*, the most common appearance of reptilian tooth-bone interface is presented with typical asymmetrical pleurodont teeth. B) Transversal section of *Varanus beccarii* jaw clearly displays the tissue called plicidentin, which connects the dentin of tooth to bone pedicles. C, D) *Salvator rufescens* possess different types and depth of the tooth implantation across the jawbone.

Figure 5: Wall thickness analysis of reptilian teeth and adjacent jaws.

A) *Anolis equestris:* The thickest area on anolis skull is localized along the upper part of the lower jaw suggesting its importance as a support during food processing. B) Sagittal section through the midline of the skull in *Anolis equestris*, C) *Salvator rufescens:* In contrast to anolis, wall thickness analysis displayed the greatest tissue density in bulbous caudal teeth, which needs to be strong enough to process hard materials such as shells. D) Sagittal section through the midline of the skull of *Salvator rufescens.*

Figure 6: Segmentation and analyses of individual teeth in *Bitis gabonica*.

A) The second generation of the venomous tooth on the left side of the upper jaw was selected for wall thickness analysis. B) Detail view on the second largest segmented tooth with applied wall thickness analysis, where red color labels the thickest area of the fang and the blue the thinnest areas. The same tooth was made partially transparent to display the inner structure of the tooth. C) All of the venomous teeth on the left side of upper jaw were segmented to uncover replacement tooth generations. D) Detail view on all generations of fangs visualized in different colour. Note differences in the size and position of distal openings among individual generations.

Figure 7: Visualization of replacement teeth in *Caiman crocodilus*.

A) Localization of individual teeth in the jaw with one segmented tooth. For precise morphology description, wall thickness analysis was applied. Orange color labels the thickest areas of the tooth. B) Segmented tooth without surrounding jawbone with is display by wall thickness analyses. Horizontal section through the analyzed tooth revealed new generation of teeth located inside of functional tooth. C) Replacement tooth is labelled in green color and functional tooth is translucent to visualize the position of next generation formation.

Figure 8: Palatal view on the upper jaw and transversal sections through the palatal teeth

A) *Chamaeleo calyptratus:* palatal view of 3D render image, B) *Chamaeleo calyptratus:* transversal section through the skull defined by the red plane indicated in 3D render displays the absence of palatal teeth, C) *Scincus scincus:* palatal view of 3D render image uncovers two pairs of palatal teeth located on the pterygoid bone, D) *Scincus scincus:* transversal section through the skull defined by the red plane indicated in 3D render visualize the palatal teeth, yellow arrow indicates the location of teeth, E) *Timon lepidus:* palatal view of 3D render image displays long row of palatal teeth, F) *Timon lepidus:* transversal section through the skull defined by the red plane indicated in 3D render demonstrates the palatal teeth. Yellow coloured arrow in D) and F) indicates the location of palatal teeth.

Figure 9: Arrangement of bones contributing to the palate in *Python regius* and *Scincus scincus.*

A) Palatal view on the upper jaw of the *Python regius,* B) Sagittal view on the bones supporting the palate in *Python regius,* C) Palatal view on the upper jaw of the *Scincus scincus,* D) Sagittal view on the bones supporting the palate in *Scincus scincus.*

Individual skeletal elements were segmented, and they were labelled in different colours. Orange – premaxillary bone, yellow – vomer, green – palatal bone, pink – pterygoid, blue – ectopterygoid. The bones of skull surrounding the segmented palatal bones were made semi-transparent to improve the display of the analysed areas.

Figure 10: Segmented bones contributing to the palate in selected reptiles

A) *Python regius,* B) *Scincus scincus.* Palatal view on individual segmented skeletal elements in both presented species: orange – premaxillary bone, yellow – vomer, green – palatal bone, pink – pterygoid, blue – ectopterygoid.

Figure 11: 3D printed skull of the *Chamaeleo calyptratus* from generated stl model.

A) 3D stl model of the scanned *Chamaeleo calyptratus* skull generated in VG Studio MAX, B) 3D printed model of the skull. For the purpose of 3D print, the generated stl model was 3.5x magnified. The PRUSA MK3S printer with the PLA filament and 0.15 mm printing layer was used to create the model, the total print time was 40 hours.

VIDEOS

Video 1: *Salvator rufescens* in 3D view while using wall thickness analyses

Video 2: 3D view of individual fang teeth in *Bitis gabonica*

Video 3: 3D visualization of replacement teeth in *Caiman crocodilus*

Video 4: *Scincus scincus* in 3D view with focus on segmented palatal elements

 Click here to access/download;Table;Tables Oct2021 .docx

Table 1: Scanning parameters for individual analysed animals

**Sample**	**Voltage [kV]**	**Current [μA]**	**Timing [ms]**	**Images**	**Filter [mm]**	**Time [min]**	**Resolution [μm]**
Anolis equestris	60	230	500	2400	0.2 Al	100	24
Bitis gabonica	60	230	500	2600	0.2 Al	105	19
Caiman crocodilus	60	230	500	2000	0.2 Al	75	45
Chamaeleo calyptratus	60	230	500	2600	0.2 Al	90	26
Timon lepidus	60	230	500	2600	0.2 Al	105	18
Paroedura picta	60	200	750	2500	0.2 Al	100	12.5
Pogona vitticeps	60	230	500	2600	0.2 Al	110	25
Python regius	60	230	500	2500	0.2 Al	105	24.5
Salvator rufescens	60	230	500	2600	0.2 Al	105	48
Scincus scincus	60	230	500	2100	0.2 Al	80	13
Varanus beccarii	60	230	500	2600	0.2 Al	110	28

Table 2: Volumes of analysed skulls

Volume (mm^3)	Skull	Maxilla	Mandible
Anolis equestris	870	584	286
Bitis gabonica	232	181	51
Caiman crocodilus	6409	4108	2301
Chamaeleo calyptratus	1441	1062	379
Timon lepidus	197	133	64
Paroedura picta	113	81	32
Pogona vitticeps	2236	1384	852
Python regius	1817	1308	509
Salvator rufescens	10869	6232	4637
Scincus scincus	108	71	37
Varanus beccarii	1130	757	373

Table 3: List of analyzed chameleon embryo samples

Sample	Date of clutch laying	Date of embryo collection	Age of embryo (days)	Weight of the egg (g)	Weight of the embryo (g)
Stage 1 (CH379)	12.01.2019	20.5.2019	128	1.23	0.25
Stage 2 (CHM313)	08.05.2018	31.08.2018	115	1.31	0.37
Stage 3 (CHM389)	12.01.2019	27.5.2019	135	1.77	0.4
Stage 4 (CHM397)	12.01.2019	6.6.2019	145	1.47	0.44
Stage 5 (CHM327)	21.06.2018	14.11.2018	146	1.81	0.65
Stage 6 (CHM351)	21.6.2018	10.12.2018	173	1.38	0.82

Table 4: Scanning parameters for individual analysed pre-hatching embryos

Sample	Voltage [kV]	Current [μA]	Timing [ms]	Images	Filter [mm]	Time [min]	Resolution [μm]
Stage 1 (CH379)	60	200	600	2500	0.2 Al	105	2.5
Stage 2 (CHM313)	60	200	600	2400	0.2 Al	105	3.5
Stage 3 (CHM389)	60	200	600	2400	0.2 Al	105	3
Stage 4 (CHM397)	60	200	600	2400	0.2 Al	105	3
Stage 5 (CHM327)	60	200	600	2200	0.2 Al	85	3
Stage 6 (CHM351)	60	200	600	2300	0.2 Al	85	3.5

Figure1

Click here to access/download;Figure;fig 1 v2.tif

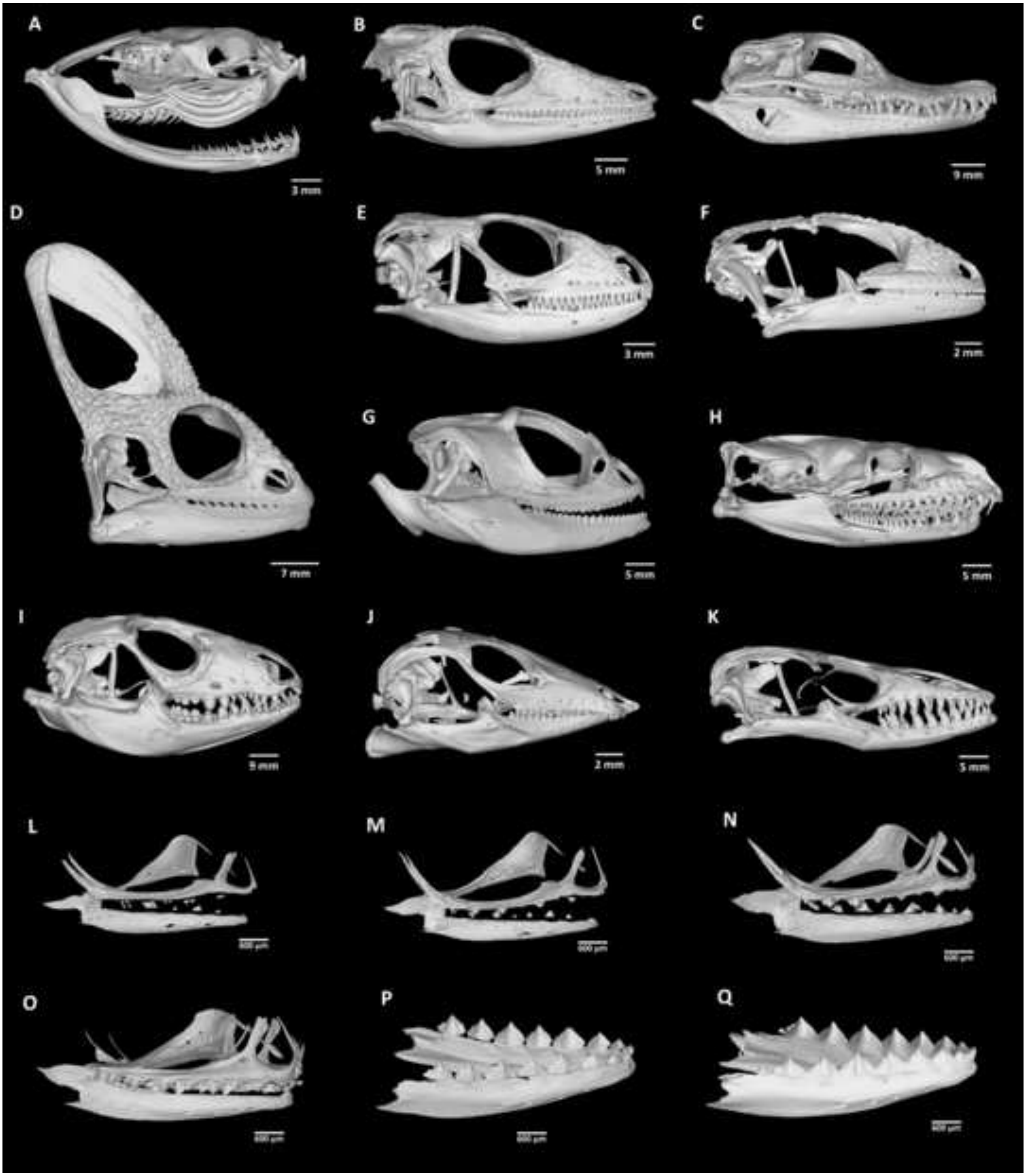

Figure2 Click here to access/download;Figure;fig 2 v2.tif

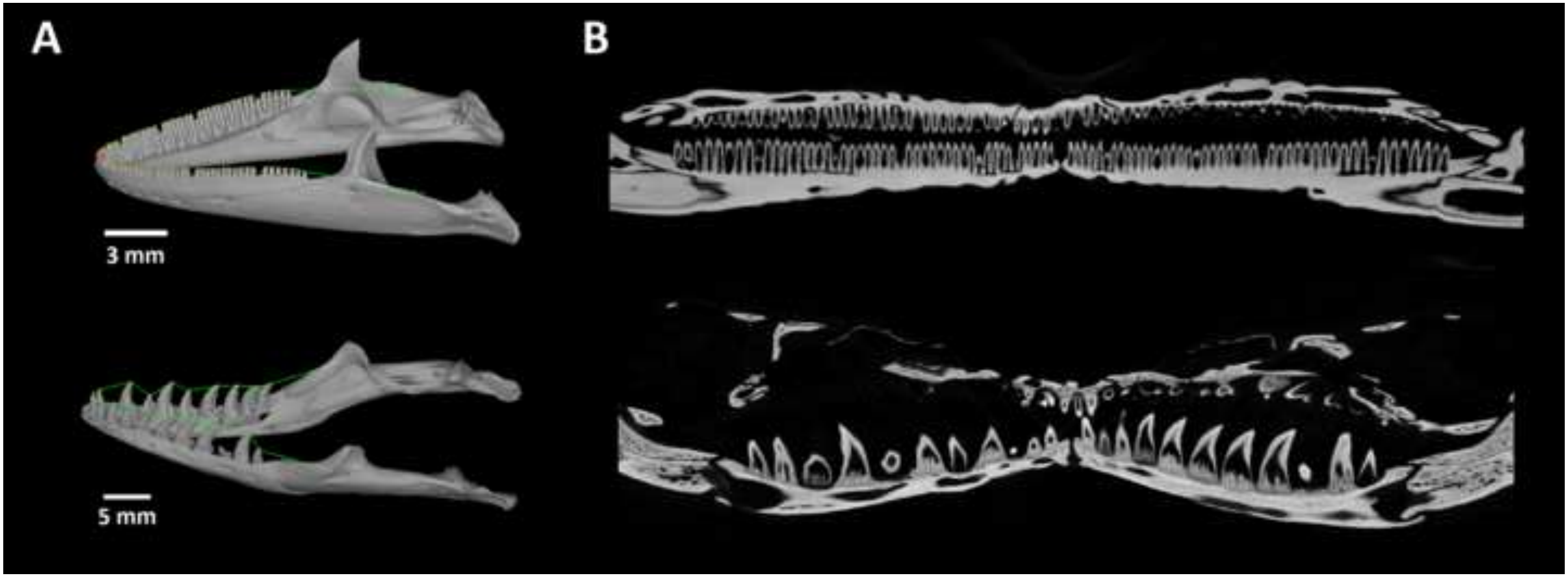

Figure3

Click here to access/download;Figure;fig 3 v2.tif

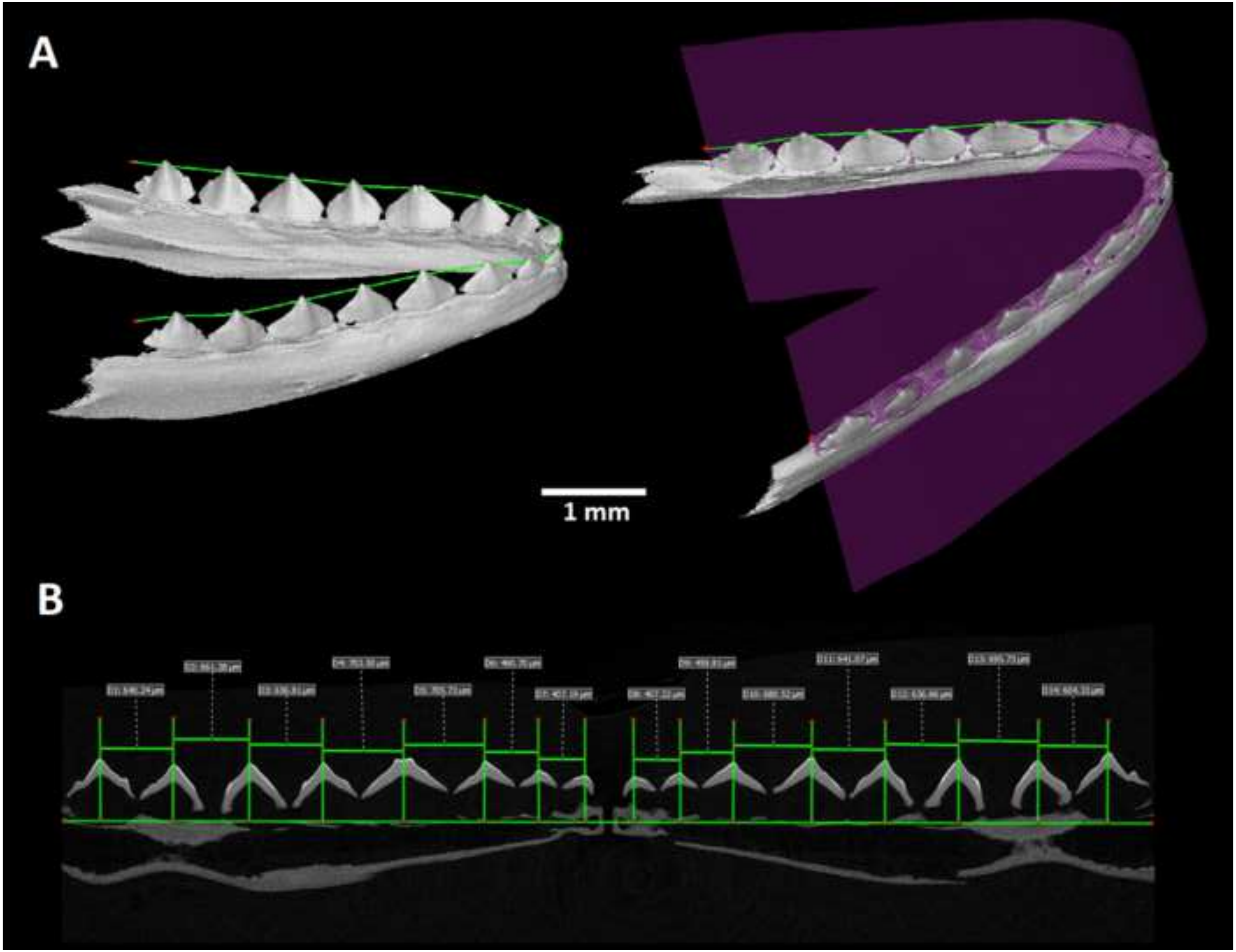

Click here to access/download;Figure;fig 4 v2.tif

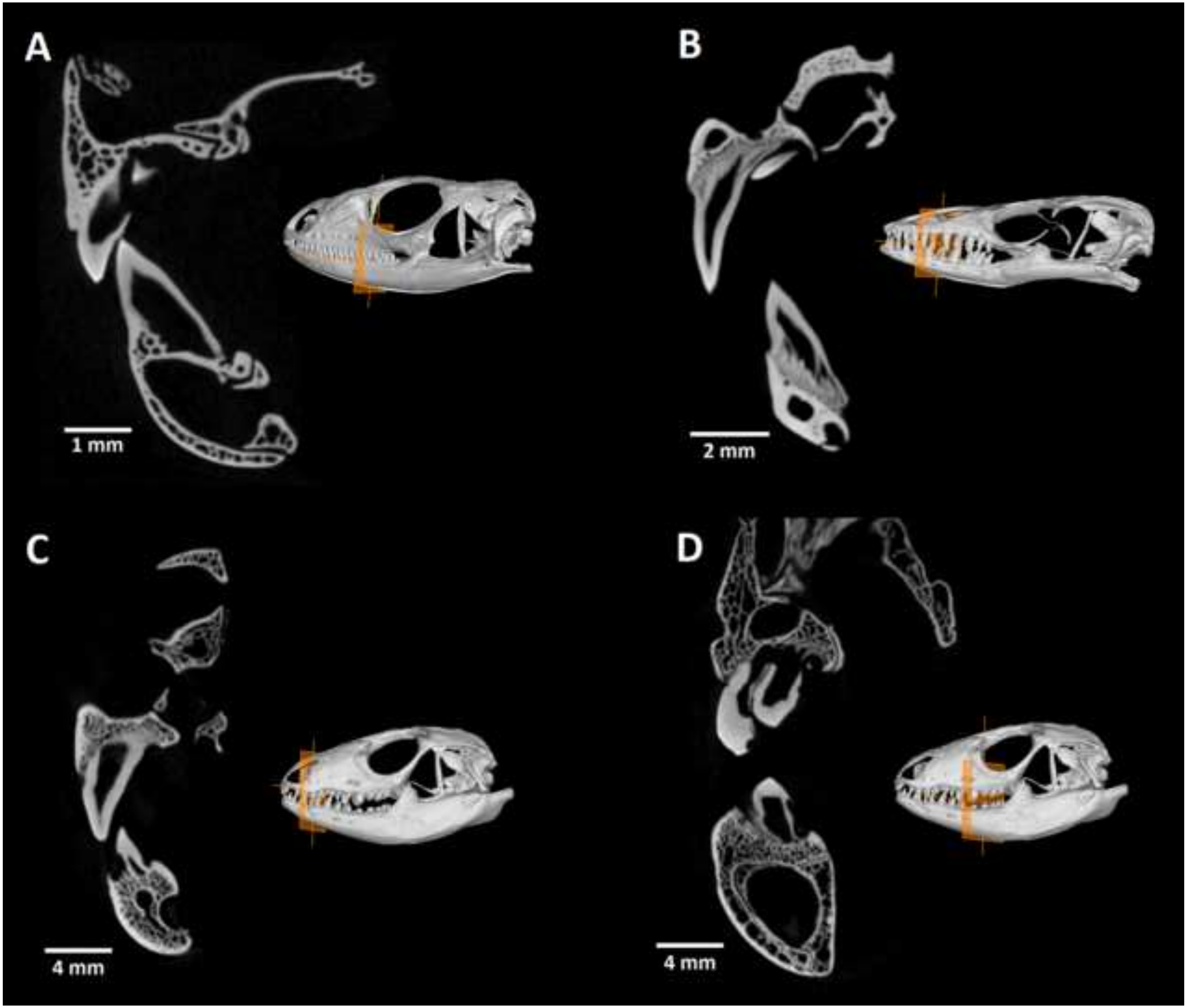

Figure5

Click here to access/download;Figure;fig 5 v2.tif

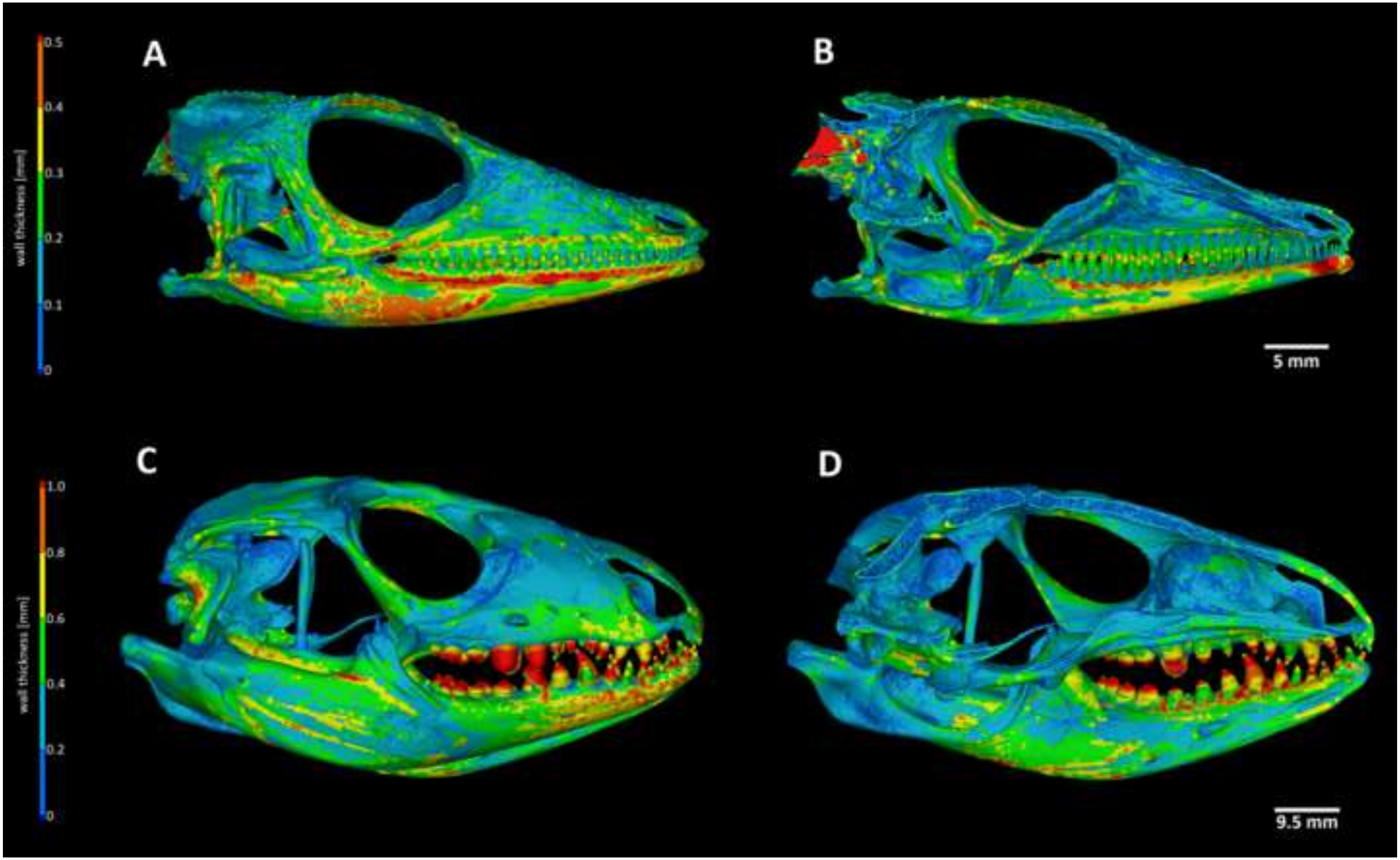

 Click here to access/download;Figure;fig 6 v2.tif

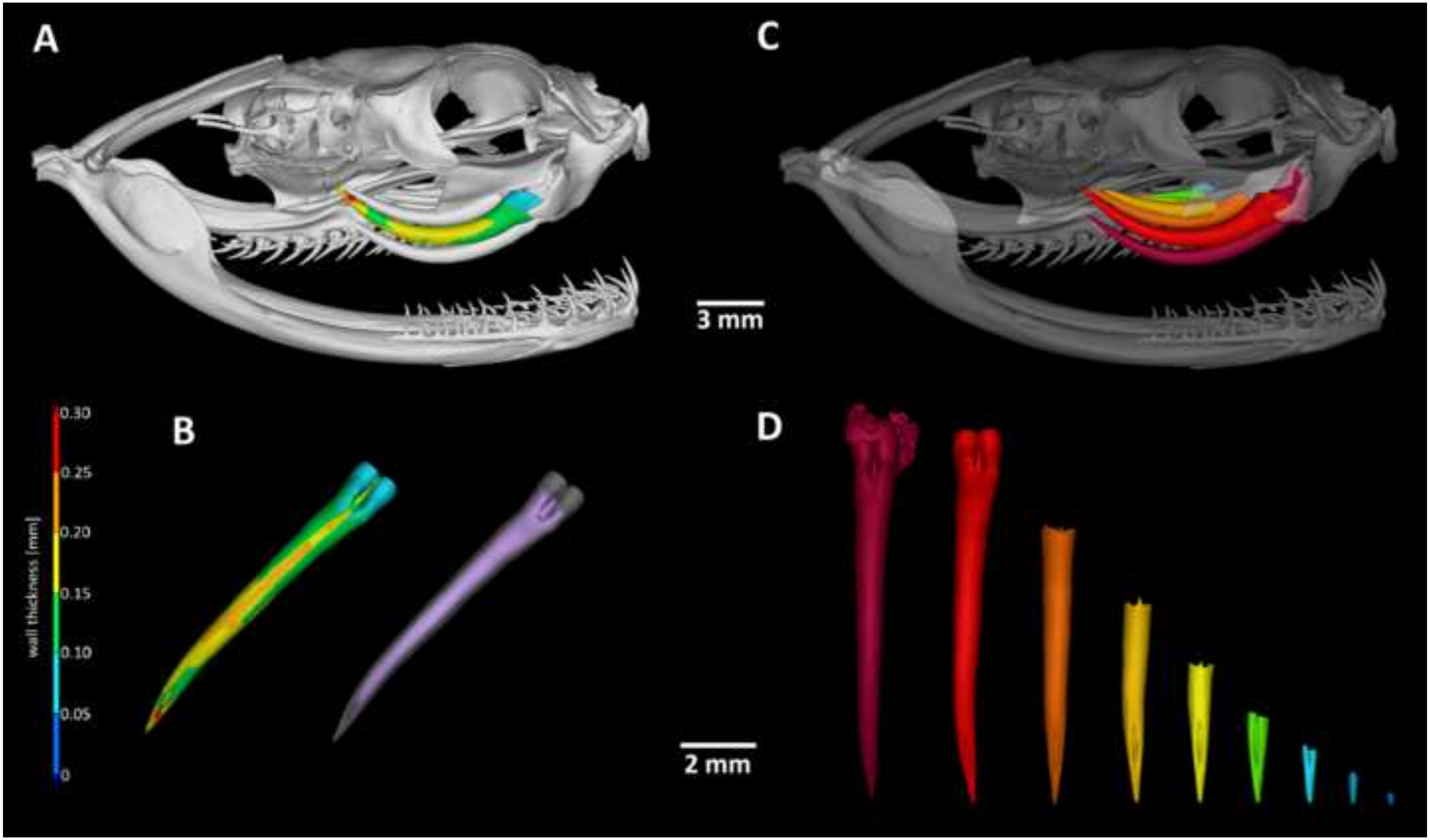

 Click here to access/download;Figure;fig 7 v2.tif

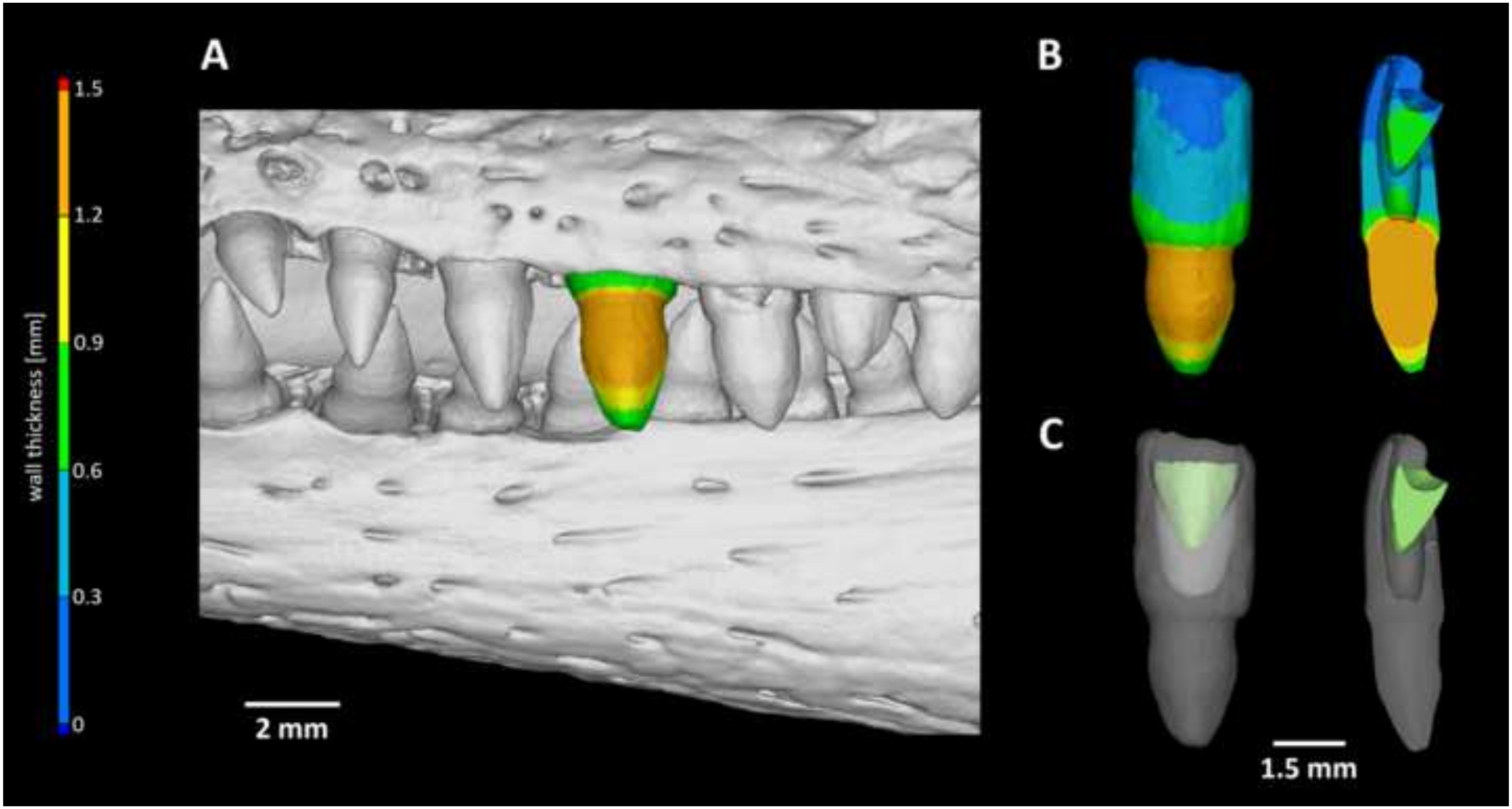

Figure8

Click here to access/download;Figure;fig 8 v2.tif

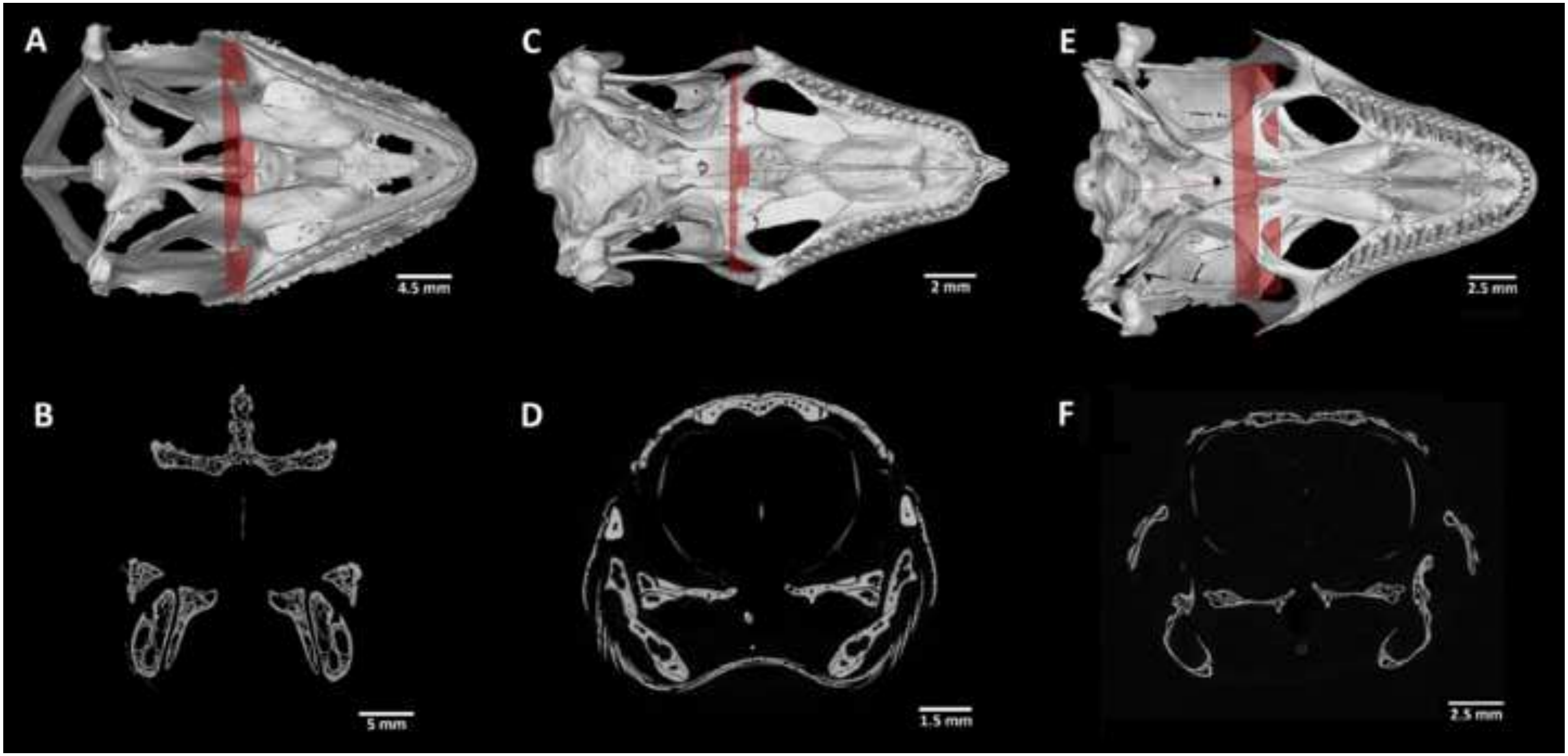

Figure9

Click here to access/download;Figure;fig 9 v2.tif

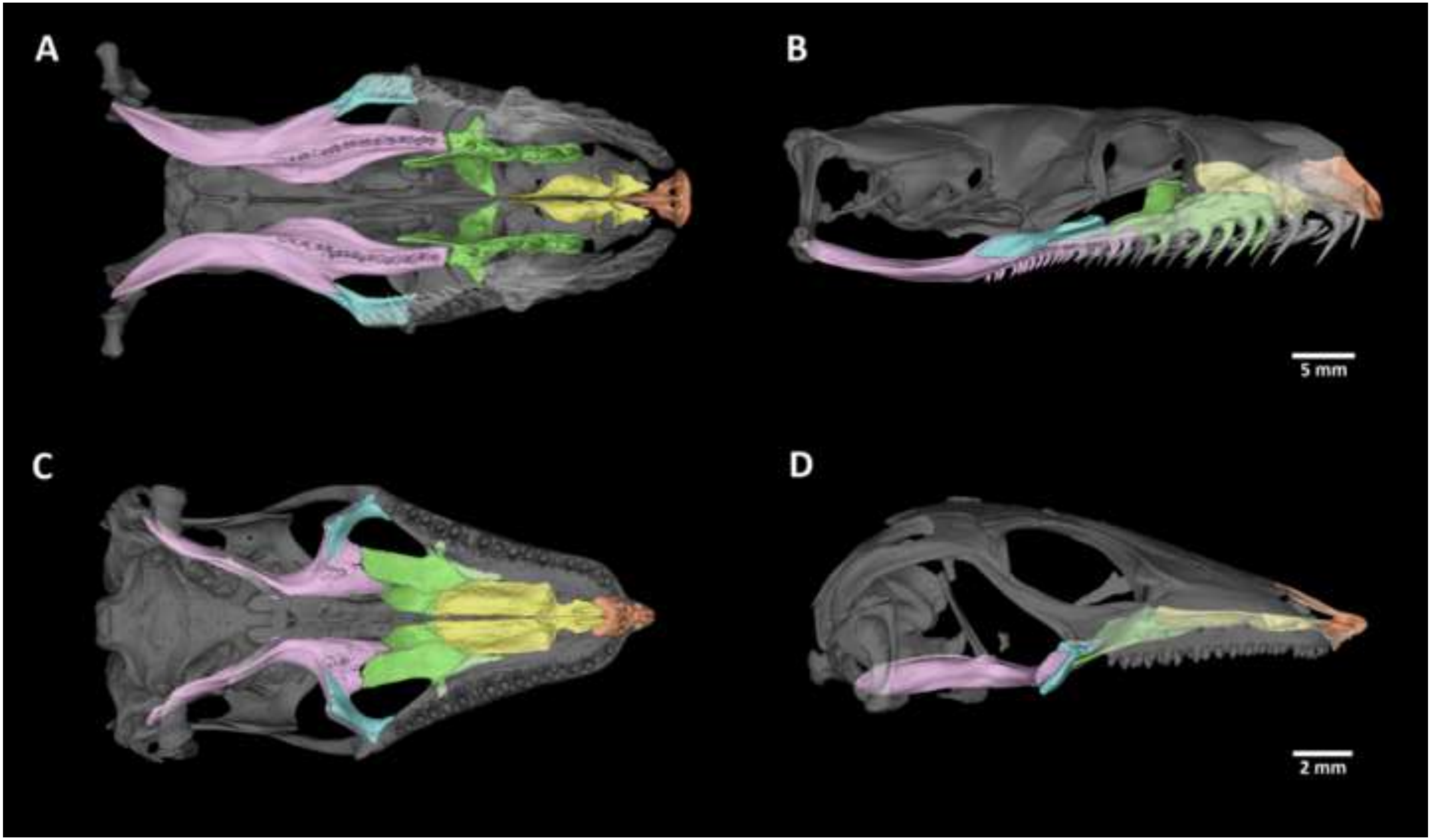

Figure10 Click here to access/download;Figure;fig 10 v2.tif

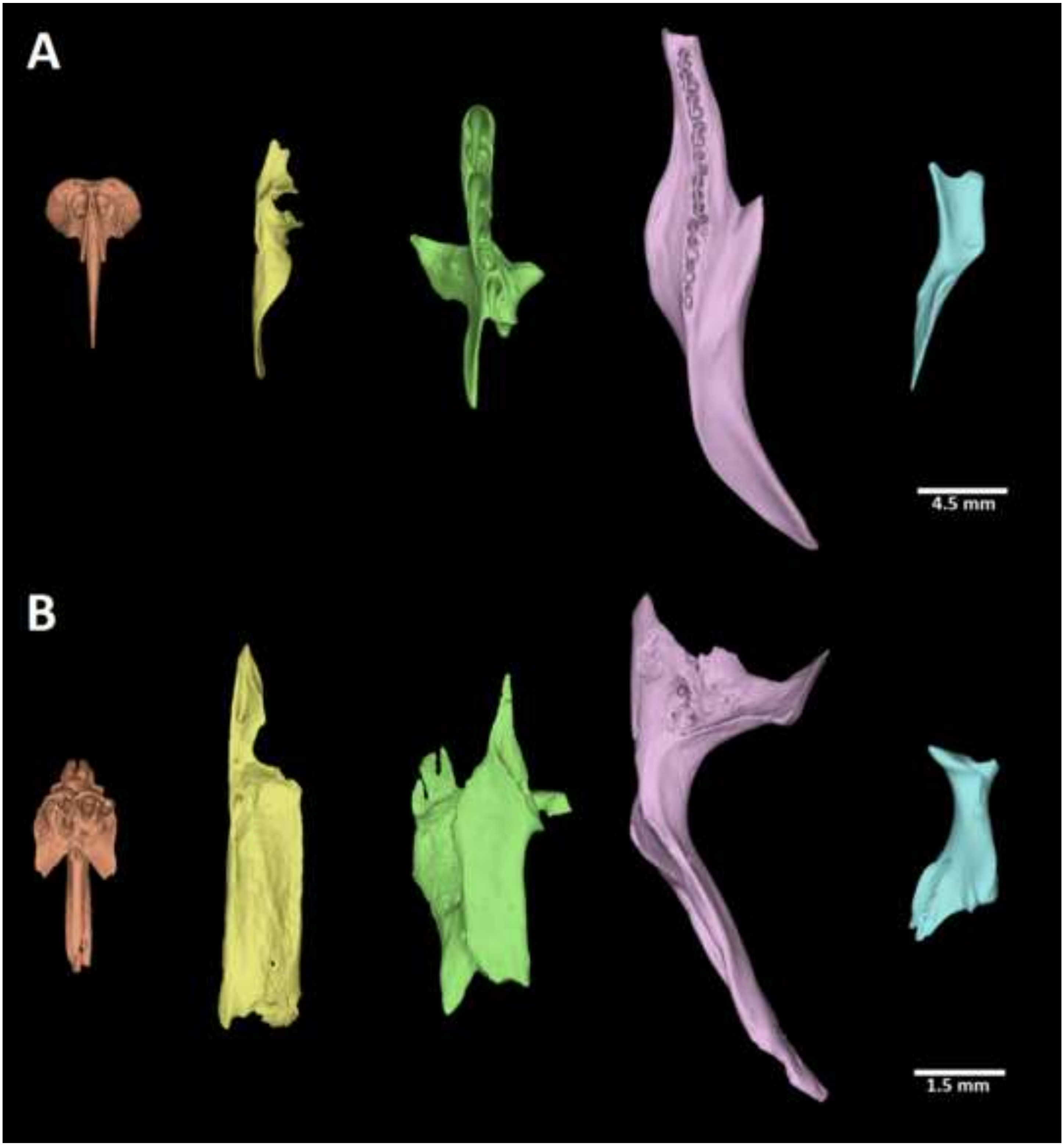

Figure11

Click here to access/download;Figure;fig 11 v2.tif

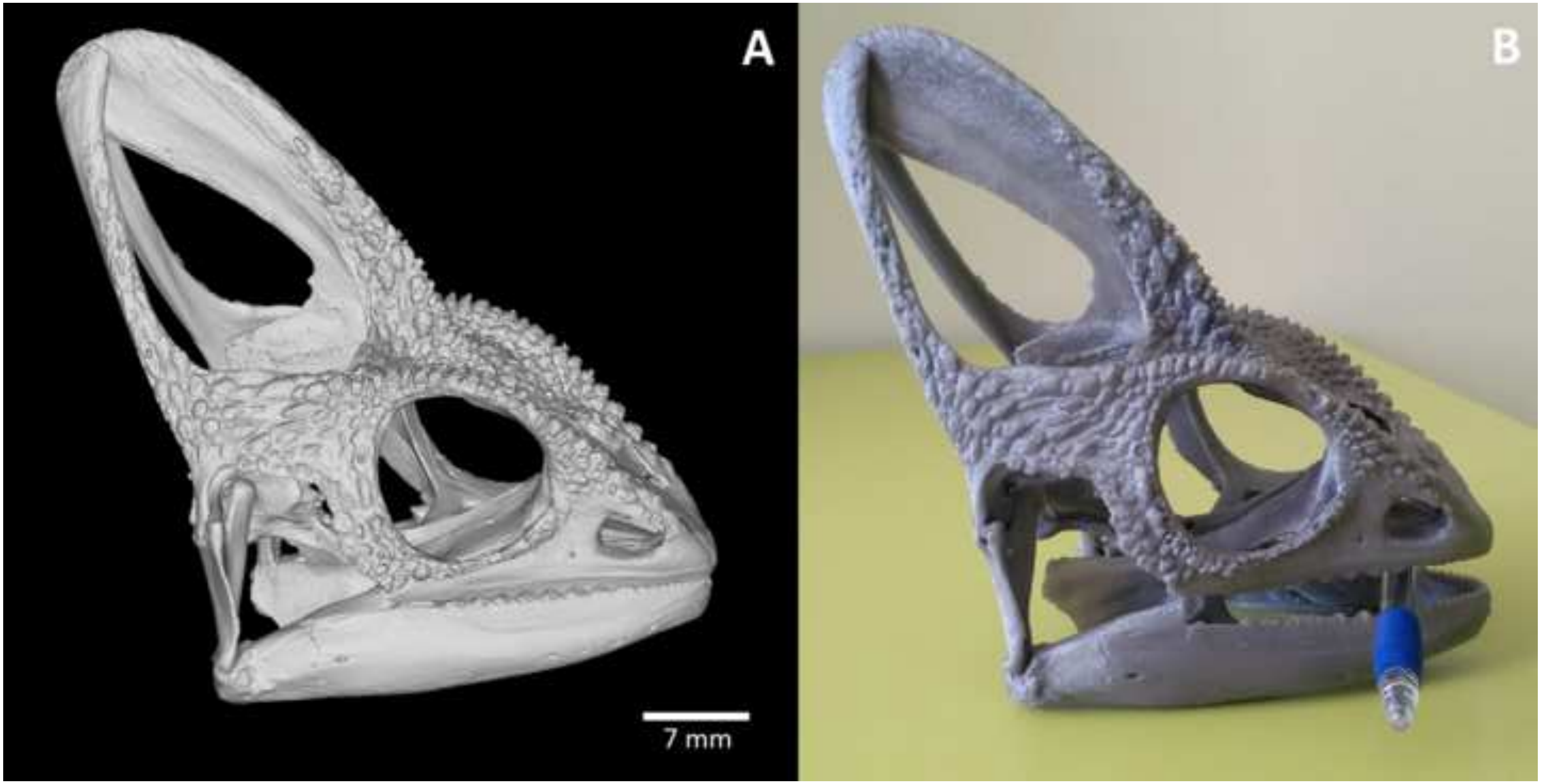

Video 1

Click here to access/download

Supplementary Material

Video 1 salvator.wmv

Video 2

Click here to access/download
Supplementary Material
Video 2 bitis.wmv

Video 3

Click here to access/download
Supplementary Material
Video 3 crocodile.wmv

Video 4

Click here to access/download

Supplementary Material

Video 4 scink.wmv

Supplementary file

Click here to access/download
Supplementary Material
varanus beccarii.wmv
