# Supplementary material for: X-ray microtomography imaging of craniofacial hard tissues in selected reptile species with different types of dentition
Source: Gigascience. 2022 Mar 7;11:giac016. doi: 10.1093/gigascience/giac016 (PMC8900495; doi:10.1093/gigascience/giac016)
Supplement: giac016_Response_to_Reviewer_Comments_Revision_1 [file giac016_Response_to_Reviewer_Comments_Revision_1.pdf]

Author's Response To Reviewer Comments

Close

Editor:
In addition, it was brought to my attention that the following two relevant recent publications should be cited and briefly discussed in your data note:

Watanabe et al. 2019, PNAS
Ecomorphological diversification in squamates from conserved pattern of cranial integration

Lafuma et al. 2021, Nature Communications
Multiple evolutionary origins and losses of tooth complexity in squamates

Please also ensure that your revised manuscript conforms to the journal style, which can be found in the Instructions for Authors on the journal homepage. In particular, please structure your abstract ("background, findings, conclusions") and use numbered references.
ANSWER:
In revised version, we included recommended references in the text of manuscript as well as reference list.
Abstract was adjusted to journal format.
References were switched to numbered format.

Reviewer 1
Reviewer #1: This data note presents microCT scans of a variety of reptiles focusing on tooth morphology. It is very interesting and well presented, with nice images and high quality data. A note on the data - the image stacks I viewed are good quality but the STL's I checked all have something wrong, I could not open these, you will have to check these. The acquisition is well reported and I think the work can be published. I think the authors might find it interesting that previous work in Gigascience also presented microCT scans of a range of venomous snake fangs (but not the gaboon viper), it might be interesting to reference this https://doi.org/10.1093/gigascience/gix126

ANSWER:
Thank you for your kind comments.
We hope STL´s are working now, we also tried to open them from several computers, we have not notice further difficulties. But it is possible that there can be some troubles while using computer with lower memories as we found this as limitation of usage. In case of any further difficulties please could you contact Michaela Kavkova (Michaela.Kavkova@ceitec.vutbr.cz) to find where these difficulties arise.
Mentioned reference is now included in the text as well as reference list.

Reviewer 2
* Timon lepidus (files are named Lacerta lepida)
ANSWER:
In the text, we are now uniformly call this species as Timon lepidus, but we also included older still commonly used name Lacerta lepida in the manuscript. The database did not allow us to rename files to Timon lepidus but we can do loading of these files again in case reviewer and editor would prefer it.

Minor comment 1
According to Table 3, the Stage 1 Chamaeleo calyptratus embryo is older than the Stage 2 Chamaeleo calyptratus embryo. This would suggest that staging is based on alternative criteria than age, such as the weight of the embryo. Can the authors please clarify what criteria were used to stage the chameleon embryos?
ANSWER:
Our staging system is based both on the age of the embryos and also the weight of each individual. By such, we can easily compare also the animals from different clutches since they can develop in different

speed. This was also the case of chameleons named stage 1 and chameleon stage 2 where the animal was younger, but the embryo was heavier (0.37 g) and more developed. The animal came from different clutch in comparison to stage 1 chameleon with the weight of only 0.25 g and it was less developed. In addition to length of pre-hatching development and their weight which is more corresponding to actual developmental stage, we also evaluate morphological features, which help us to better determine the level of development in oviparous reptiles where the length of development varies between clutches as well as inside individual clutch.

Minor comment 2
For the Stage 6 Chamaeleo calyptratus embryo, a 3D surface reconstruction (STL format) is provided of segmented dentition. Can the authors please clarify whether they will additionally be submitting a 3D surface reconstruction (STL format) of Stage 6 embryo craniofacial skeletal components to the GigaScience Database?
ANSWER:
Unfortunately, we do not have available scans of the upper jaw. For this larger animal, we wanted to have similar resolution scans to the smallest animals therefore we had to perform scanning of just proportion of head. We are aware this weakness and impossibility to analyze upper jaw, but we hoped that readers can use at least lower jaw scans for teeth and certain skeletal analyses.

Thank you for your kind comments.

Close
