# Supplementary material for: X-ray microtomography imaging of craniofacial hard tissues in selected reptile species with different types of dentition
Source: Gigascience. 2022 Mar 7;11:giac016. doi: 10.1093/gigascience/giac016 (PMC8900495; doi:10.1093/gigascience/giac016)
Supplement: giac016_Response_to_Reviewer_Comments_Revision_2 [file giac016_Response_to_Reviewer_Comments_Revision_2.pdf]

Author's Response To Reviewer Comments

Close

Dear editor,
we followed instruction and made all necessary changes.
Thank you for your help.
With best regards
Marcela Buchtova

Close
