# Supplementary material for: X-ray microtomography imaging of craniofacial hard tissues in selected reptile species with different types of dentition
Source: Gigascience. 2022 Mar 7;11:giac016. doi: 10.1093/gigascience/giac016 (PMC8900495; doi:10.1093/gigascience/giac016)
Supplement: giac016_Reviewer_2_Report_Original_Submission — Chris Armit -- 12/1/2021 Reviewed [file giac016_Reviewer_2_Report_Original_Submission.pdf]

Reviewer Report

Title: X-ray microtomography imaging of craniofacial hard tissues in selected reptile species with different types of dentition

Version: Original Submission **Date:** 12/1/2021

Reviewer name: Chris Armit

Reviewer Comments to Author:

This exceptional Data Note showcases an impressive set of microCT 3D reconstructions of reptile skulls that includes segmentations of skeletal components and dentition, enabling comparative analysis of craniofacial anatomy in reptilia. The specimens are of tissue-level resolution with the difference in voxel size being directly linked to the physical dimensions of the specimen. Of note, the microCT scans are of hard tissue and so there was no need for staining with contrast agents, which is only needed to improve the contrast of soft tissues. The 3D models are of very high quality, and individual skeletal components - including premaxilla, vomer, palatal bone, pterygoid, ectopterygoid - were segmented and their volumes were calculated. The sclerotic ring surrounding the eye is additionally visible in 3D reconstructions of Bearded Dragon (Pogona vitticeps) and Ocelot Gecko (Paroedura picta). For analysis of single-tooth morphology, dentition was segmented using thresholding operations and a 3D brush, and this approach was used to explore dental pulp morphology in venomous fangs of Bitis gabonica. In addition, the authors used polylines to determine surface structure of dentition and to measure tooth spacing. Furthermore, the authors provide a novel visualisation of teeth by digitally "unrolling" the jaw of reptile craniofacial 3D reconstructions to display dentition in a linear fashion.
The supporting data includes microCT image data (volumetric TIFF stack) and surface-rendered 3D reconstructions (STL format) of reptilian skulls of the following species:
* Anolis equestris
* Bitis gabonica
* Caiman crocodilus
* Chamaeleo calyptratus
* Timon Lepidus (files are named Lacerta lepida)
* Paroedura picta
* Pogona vitticeps
* Python regius
* Salvator rufescens
* Scincus scincus
* Varanus beccarii
In addition, the authors showcase a developmental time-series of multiple pre-hatching stages of the developing skull of the Veiled Chameleon (Chamaeleo calyptratus). This detailed developmental time-series of 3D models allows researchers to more accurately understand the morphological changes that are observed during reptilian craniofacial development, and the authors used this time-series of models to explore the morphological changes in tooth development in the Veiled Chameleon where ankylosis

(i.e. fusion of dentition and bone) is a developmental process.
For this developmental time-series of models, the supporting data includes microCT image data (volumetric TIFF stack) and surface-rendered 3D reconstructions (STL format) of Chamaeleo calyptratus embryos at the following stages / embryonic age:
* Chamaeleo calyptratus Stage 1 (CH379) (age 128 days)
* Chamaeleo calyptratus Stage 2 (CHM313) (age 115 days)
* Chamaeleo calyptratus Stage 3 (CHM389) (age 135 days)
* Chamaeleo calyptratus Stage 4 (CHM397) (age 145 days)
* Chamaeleo calyptratus Stage 5 (CHM327) (age 146 days)
* Chamaeleo calyptratus Stage 6 (CHM351) (age 173 days)
This collection of 3D reconstructions is an important resource for the understanding comparative anatomy and it is my recommendation that this Data Note is accepted for publication in GigaScience.
Minor comment 1
According to Table 3, the Stage 1 Chamaeleo calyptratus embryo is older than the Stage 2 Chamaeleo calyptratus embryo. This would suggest that staging is based on alternative criteria than age, such as the weight of the embryo. Can the authors please clarify what criteria were used to stage the chameleon embryos?
Minor comment 2
For the Stage 6 Chamaeleo calyptratus embryo, a 3D surface reconstruction (STL format) is provided of segmented dentition. Can the authors please clarify whether they will additionally be submitting a 3D surface reconstruction (STL format) of Stage 6 embryo craniofacial skeletal components to the GigaScience Database?

Level of Interest

Please indicate how interesting you found the manuscript: Choose an item.

Quality of Written English

Please indicate the quality of language in the manuscript: Choose an item.

Declaration of Competing Interests

Please complete a declaration of competing interests, considering the following questions:

- Have you in the past five years received reimbursements, fees, funding, or salary from an organisation that may in any way gain or lose financially from the publication of this manuscript, either now or in the future?
- Do you hold any stocks or shares in an organisation that may in any way gain or lose financially from the publication of this manuscript, either now or in the future?

- Do you hold or are you currently applying for any patents relating to the content of the manuscript?
- Have you received reimbursements, fees, funding, or salary from an organization that holds or has applied for patents relating to the content of the manuscript?
- Do you have any other financial competing interests?
- Do you have any non-financial competing interests in relation to this paper?

If you can answer no to all of the above, write 'I declare that I have no competing interests' below. If your reply is yes to any, please give details below.

I work for GigaScience where I perform checks on the quality of image data and the image analysis approach. My funding is not dependent on the outcome of this review, and I declare that I have no competing interests.

I agree to the open peer review policy of the journal. I understand that my name will be included on my report to the authors and, if the manuscript is accepted for publication, my named report including any attachments I upload will be posted on the website along with the authors' responses. I agree for my report to be made available under an Open Access Creative Commons CC-BY license (http://creativecommons.org/licenses/by/4.0/). I understand that any comments which I do not wish to be included in my named report can be included as confidential comments to the editors, which will not be published.

Choose an item.

To further support our reviewers, we have joined with Publons, where you can gain additional credit to further highlight your hard work (see: https://publons.com/journal/530/gigascience). On publication of this paper, your review will be automatically added to Publons, you can then choose whether or not to claim your Publons credit. I understand this statement.

Yes Choose an item.
