# Supplementary material for: X-ray microtomography imaging of craniofacial hard tissues in selected reptile species with different types of dentition
Source: Gigascience. 2022 Mar 7;11:giac016. doi: 10.1093/gigascience/giac016 (PMC8900495; doi:10.1093/gigascience/giac016)
Supplement: giac016_Reviewer_2_Report_Revision_1 — Chris Armit -- 12/21/2021 Reviewed [file giac016_Reviewer_2_Report_Revision_1.pdf]

Reviewer Report

Title: X-ray microtomography imaging of craniofacial hard tissues in selected reptile species with different types of dentition

Version: Revision 1 **Date:** 12/21/2021

Reviewer name: Chris Armit

Reviewer Comments to Author:

I thank the authors for their response and I confirm that the authors have addressed my question relating to the staging system used for the Chamaeleo calyptratus embryos. In addition, the authors have explained that, for the larger Stage 6 chameleon embryo, the scan of the upper jaw is missing and that this was necessary to ensure a high-resolution scan of the lower jaw. This is a perfectly reasonable explanation and the authors are to be commended for providing a time-series of Chamaeleo calyptratus embryonic skeletal components with a focus on craniofacial development. I see immense reuse potential in the developmental time-series of chameleon 3D models, and from a comparative anatomy perspective I think that the chameleon embryo microCT image data with the exploration of tooth attachment are a great addition to the collection of adult reptilian skulls with dentition that are also presented in this Data Note.
The manuscript is well written, and the methods are appropriate for this anatomical and morphological study. In addition, I can confirm that the surface reconstructions (STL files) can be opened using MeshLab. It is my recommendation that this Data Note is accepted for publication in GigaScience. I further recommend that the GigaScience Database provide interactive 3D visualisations of the surface reconstruction 3D models as a means of showcasing this exceptional microCT dataset.

Level of Interest

Please indicate how interesting you found the manuscript: Choose an item.

Quality of Written English

Please indicate the quality of language in the manuscript: Choose an item.

Declaration of Competing Interests

Please complete a declaration of competing interests, considering the following questions:

- Have you in the past five years received reimbursements, fees, funding, or salary from an organisation that may in any way gain or lose financially from the publication of this manuscript, either now or in the future?

- Do you hold any stocks or shares in an organisation that may in any way gain or lose financially from the publication of this manuscript, either now or in the future?
- Do you hold or are you currently applying for any patents relating to the content of the manuscript?
- Have you received reimbursements, fees, funding, or salary from an organization that holds or has applied for patents relating to the content of the manuscript?
- Do you have any other financial competing interests?
- Do you have any non-financial competing interests in relation to this paper?

If you can answer no to all of the above, write 'I declare that I have no competing interests' below. If your reply is yes to any, please give details below.

I work for GigaScience where I perform checks on the quality of image data and the image analysis approach. My funding is not dependent on the outcome of this review, and I declare that I have no competing interests.

I agree to the open peer review policy of the journal. I understand that my name will be included on my report to the authors and, if the manuscript is accepted for publication, my named report including any attachments I upload will be posted on the website along with the authors' responses. I agree for my report to be made available under an Open Access Creative Commons CC-BY license (http://creativecommons.org/licenses/by/4.0/). I understand that any comments which I do not wish to be included in my named report can be included as confidential comments to the editors, which will not be published.

Choose an item.

To further support our reviewers, we have joined with Publons, where you can gain additional credit to further highlight your hard work (see: https://publons.com/journal/530/gigascience). On publication of this paper, your review will be automatically added to Publons, you can then choose whether or not to claim your Publons credit. I understand this statement.

Yes Choose an item.
